# Chemical Perturbation of Chloroplast-Related Processes Affects Circadian Rhythms of Gene Expression in *Arabidopsis*: Salicylic Acid Application Can Entrain the Clock

**DOI:** 10.3389/fphys.2020.00429

**Published:** 2020-06-18

**Authors:** Koumis Philippou, Amanda M. Davis, Seth J. Davis, Alfredo Sánchez-Villarreal

**Affiliations:** ^1^Department of Plant Developmental Biology, Max Planck Institute for Plant Breeding Research, Cologne, Germany; ^2^Department of Biology, University of York, York, United Kingdom; ^3^Key Laboratory of Plant Stress Biology, School of Life Sciences, Henan University, Kaifeng, China

**Keywords:** circadian clock, *Arabidopsis*, luciferase imaging, metabolic inputs, entrainment, stress signaling, salicylic acid, redox

## Abstract

The plant circadian system reciprocally interacts with metabolic processes. To investigate entrainment features in metabolic–circadian interactions, we used a chemical approach to perturb metabolism and monitored the pace of nuclear-driven circadian oscillations. We found that chemicals that alter chloroplast-related functions modified the circadian rhythms. Both vitamin C and paraquat altered the circadian period in a light-quality-dependent manner, whereas rifampicin lengthened the circadian period under darkness. Salicylic acid (SA) increased oscillatory robustness and shortened the period. The latter was attenuated by sucrose addition and was also gated, taking place during the first 3 h of the subjective day. Furthermore, the effect of SA on period length was dependent on light quality and genotype. Period lengthening or shortening by these chemicals was correlated to their inferred impact on photosynthetic electron transport activity and the redox state of plastoquinone (PQ). Based on these data and on previous publications on circadian effects that alter the redox state of PQ, we propose that the photosynthetic electron transport and the redox state of PQ participate in circadian periodicity. Moreover, coupling between chloroplast-derived signals and nuclear oscillations, as observed in our chemical and genetic assays, produces traits that are predicted by previous models. SA signaling or a related process forms a rhythmic input loop to drive robust nuclear oscillations in the context predicted by the *zeitnehmer* model, which was previously developed for *Neurospora*. We further discuss the possibility that electron transport chains (ETCs) are part of this mechanism.

## Introduction

Stress events often occur at predictable times of the day given the environmentally rhythmic cycling of light, temperature, and humidity. Within these cycles, light causes the accumulation of reactive oxygen species (ROS) (Pitzschke et al., 2006), while pathogen invasion is often favored at a given time of day (Shin et al., 2012; Korneli et al., 2014; Karapetyan and Dong, 2018; Li et al., 2018; Zhang et al., 2019). These perturbations often elicit various types of oxidative bursts (Karapetyan and Dong, 2018; Zhang et al., 2019). Given the predictable, timed nature of these abiotic and biotic stressors, the plant circadian clock provides timed sensitivity resistance to such agents. This 24-h oscillator serves to prime a plant to be most capable of resisting stress when it is most likely to be encountered (Covington et al., 2008; Sánchez et al., 2011; Fornara et al., 2015; Grundy et al., 2015). Whether these stress agents themselves feedback to tune the oscillator is still much less understood. In *Arabidopsis*, transcriptional/translational oscillations (TTOs) form feedback loops thought to be the central circadian oscillator that drives rhythmic gene expression (Bujdoso and Davis, 2013; Staiger et al., 2013; Anwer et al., 2014; Oakenfull and Davis, 2017; McClung, 2019; Webb et al., 2019). Initially, the core circadian clock was regarded as the feedback mechanism between the two morning-expressed MYB transcription factors CIRCADIAN CLOCK ASSOCIATED 1 (CCA1) and LATE ELONGATED HYPOCOTYL (LHY) and the night-phased TIMING OF CAB EXPRESSION 1 (TOC1), also known as PSEUDO RESPONSE REGULATOR 1 (PRR1) (Alabadí et al., 2001). Respective single mutants display a short-period phenotype, and rhythmicity is arrested in the triple mutant (Ding et al., 2007). Computational approaches that aimed to introduce photoperiodic perception and reconcile accumulated experimental findings led to more complex models that comprised additional TTO loops (Locke et al., 2005, 2006; Bujdoso and Davis, 2013). These models incorporated the post-transcriptional and the post-translational regulation of CCA1, LHY, TOC1, PRR9, PRR7, and GIGANTEA (GI) (Locke et al., 2006; Zeilinger et al., 2006; Pokhilko et al., 2010; Bujdoso and Davis, 2013) and the EVENING COMPLEX (EC) comprised by EARLY FLOWERING 3 (ELF3), ELF4, and LUX ARRHYTHMO (LUX) (Nusinow et al., 2011; Herrero et al., 2012; Pokhilko et al., 2012; Anwer et al., 2014; Ronald and Davis, 2017). Recently, a model with interconnected activation and repression activities within the loops including *BROTHER OF LUX ARRYTHMO* (*BOA*), *REVEILLE8* (*RVE8*), *RVE6*, *RVE4* and *LIGHT-REGULATED WD1* (*LWD1*) and *LWD2* has been proposed (McClung, 2019). This network is in constant cross-talking with plant physiology and the environment (McClung, 2019).

In 1960, Aschoff described a “rule” according to which the period of free-running oscillations changes linearly with alterations in light intensity. Aschoff’s Rule is illustrated with fluence response curves (FRCs) (Bunning, 1967). In *Arabidopsis*, photoreceptors have been linked with light input to the clock through genetic studies (Somers et al., 1998; Devlin and Kay, 2000; Somers et al., 2000, 2004; Oakenfull and Davis, 2017). From these studies, it was established that *PHYTOCHROME A* (*PHYA*) is a low-fluence photoreceptor, *PHYB* is the main red light (RL) photoreceptor, and *CRTYPTOCHROME* (*CRY1*) is the blue light (BL) photoreceptor (Somers et al., 1998). In addition to these, a BL-chromoprotein was recognized in the F-box protein ZEITLUPE (ZTL) that displays involvement in light signaling and clock protein stability (Más et al., 2003b; Kim et al., 2007; Fujiwara et al., 2008).

Entrainment to light and light-input to the clock are not identical entities (Oakenfull and Davis, 2017). For example, light input to the clock seen in the induction of *LHY* gene expression (Kim et al., 2003) is not correlated to entrainment to light pulses (Covington et al., 2001). Furthermore, entrainment can also take place in the absence of the major phytochrome and cryptochrome photoreceptors (Yanovsky et al., 2000; Strasser et al., 2010). These findings suggest that, in *Arabidopsis*, photoreceptor signaling alone cannot fully explain entrainment to light nor Aschoff’s Rule.

In cyanobacteria, it has been documented that entrainment to light does not require photoreceptors (Rust et al., 2011; Diamond et al., 2017). Light input to the clock and circadian entrainment in cyanobacteria have been connected to the redox status of the photosynthetic electron transport chain (ETC) and the redox state of the plastoquinone (PQ) pool (Mackey et al., 2011). Thus, light input could be an indirect process in supporting the entrainment without photoreceptors through metabolism as seen in *Arabidopsis*.

Metabolic oscillations have been shown to interact with TTOs in several eukaryotes, including mammals (Rutter et al., 2001; Dioum et al., 2002; Kaasik and Lee, 2004; Asher et al., 2008; Nakahata et al., 2008, 2009; O’Neill et al., 2008; Ramsey et al., 2009), plants (Panda et al., 2002; Dodd et al., 2007; James et al., 2008; Dalchau et al., 2011), fungi (Merrow et al., 1999; Yoshida et al., 2011), and protists (Bunning, 1967). In fungi, this type of interaction has been held responsible for compensation against external and metabolic perturbation (Merrow et al., 1999; Roenneberg and Merrow, 1999). Thus, the clock controls the timing of metabolism and, in return, metabolic signals set the clock.

It has been established that there is a reciprocal connection between TTOs and metabolism in higher plants (Müller et al., 2014). In *Arabidopsis*, cytosolic oscillations in cyclic adenosine diphosphate ribose and TTOs reciprocally regulate each other (Dodd et al., 2007), whereas oscillations in sugar solutes drive rhythmic gene expression (Bläsing et al., 2005). Later it was established that sugars derived from photosynthesis entrain the clock (Haydon et al., 2013), allowing for rhythmic plasticity through anabolic dawn in concordance with the photoperiod (Müller et al., 2014; Webb et al., 2019). Furthermore, perturbations in ionic conditions also have effects on clock performance (Perea-García et al., 2015). It is therefore plausible that metabolism is one driving force which is capable of performing circadian entrainment.

Metabolism can be modulated by molecules with different chemical properties. Crosstalk between metabolic networks and nuclear oscillations can be perturbed by the addition of ROS and redox-related molecules (Karapetyan and Dong, 2018). Here through a chemical biology approach, we observed effects on circadian clock parameters by paraquat, an oxidizing and uncoupling photosynthetic agent, the antioxidant vitamin C (vitC), the inhibitor of photosynthetic electron transport DCMU [3-(3,4-dichlorophenyl)-1,1-dimethylurea], and rifampicin, an inhibitor of organellar DNA-dependent RNA polymerase. Interestingly, all of these chemicals are known to alter chloroplast-driven metabolic processes. We also tested salicylic acid (SA) because it alters cellular redox status in order to trigger the cellular defense response (Mou et al., 2003), and plant innate immunity is in crosstalk with the circadian clock (Zhang et al., 2013; Korneli et al., 2014). Furthermore, even though a previous study reported that SA application did not influence circadian parameters (Hanano et al., 2006), later it was shown that SA application reinforces rhythmicity in *Arabidopsis* (Zhou et al., 2015). Here we confirm the latter effect of SA on circadian clock robustness and also show that, depending on sucrose supplementation, SA accelerates oscillations. Moreover, we show that SA affects entrainment to light–dark cycles and light pulses (parametric and non-parametric entrainment, respectively). Finally, we propose that SA signaling acts in entrainment in the context predicted by the *zeitnehmer* model, previously developed for *Neurospora* (Merrow et al., 1999; Roenneberg and Merrow, 1999), that describes rhythmic input pathways to oscillations that serve a time-keeping function.

## Results

A chemical approach was used to investigate the potential crosstalk between TTOs and metabolism in *Arabidopsis thaliana*. Redox-related chemicals were exogenously applied on seedlings and the effect of the chemicals on circadian promoter activity was monitored with the luciferase system. We tested chemicals affecting the thioredoxin and glutaredoxin systems (chlorodinitrobenzene and buthionine sulfoximine, inhibitors of thioredoxin reductase and glutathione synthesis, respectively), respiration inhibitors (antimycin A, rotenone, and salicylhydroxamic acid, which is an inhibitor of the RESPIRATORY ALTERNATIVE OXIDASE), oxidant agents such as menadione, paraquat (methylviologen), and butylhydroxyperoxide, and antioxidants, such as vitamin C and dithiocarbamate. We also tested the hormone SA, norbornadiene (inhibitor of ethylene perception), diphenyleneiodonium (inhibitor of plasma membrane NADPH oxidases involved in hypersensitive reaction during pathogen recognition), butanedione monoxime (a ROS-inducing inhibitor of cytoplasmic streaming), and photosynthesis inhibitors DCMU [3-(3,4-dichlorophenyl)-1,1-dimethylurea] and DBMIB (2,5-dibromo-3-methyl-6-isopropylbenzoquinone). This approach is similar to the chemical biology strategies previously used to investigate the *Arabidopsis* circadian clock (Toth et al., 2001; Belbin et al., 2019; Uehara et al., 2019).

**FIGURE 1:**
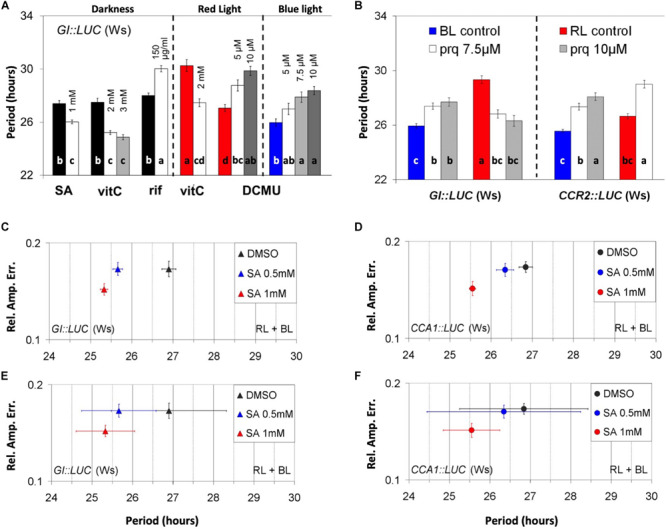
Chloroplast-related chemicals alter the parameters of nuclear oscillations. **(A)** Effect of chemicals on the circadian period under darkness (left panel), red light (middle panel) and blue light (right panel). Dark, red, and blue bars represent the respective controls. **(A)** One-way ANOVA was performed for each panel (see “Materials and Methods”). **(B)** Light quality and construct specificity effect of paraquat on period. Red and blue bars indicate respective controls. An independent ANOVA analysis was performed for each marker, *GI:LUC* (left panel) and *CCR2:LUC* (right panel). The output of the statistical analysis is shown in Supplementary Tables S1–S5). In **(A)** and **(B)**, different letters (a–d) indicate statistically significant differences between means of period. **(C–F)** Relative amplitude error of plants treated with salicylic acid (SA) using either *GI:LUC* marker **(C,E)** or *CCA1:LUC* marker **(D,F)**. The plants received two dawn events in the presence of SA, one in a white light cabinet and one in a luminometer, and then were released into free running conditions. The results shown are derived from pooled data from several experiments. Error bars represent standard error except in **(E)** and **(F)** where they represent the SD of period.

Several luciferase reporters of promoter activity were examined for their relative amplitude error (RAE) and RAE-normalized period (noPer) in a medium with or without sucrose. The chemicals that altered the noPer of rhythmic markers on a medium that contained sucrose are shown in Figure 1A (for *GI:LUC*) and Supplementary Figure S1; the statistical analyses are shown in Supplementary Tables S1–S5. The hormone SA shortened the circadian period of *GI:LUC* (Figure 1A) in the dark (DD). The aforementioned effects of SA in DD were also reproduced with *CCR2:LUC* (Supplementary Figure S1D). The antioxidant vitC shortened the circadian period of *GI:LUC* under red light (RL) and in the dark (Figure 1A) but had no effect under blue light (BL) (Supplementary Figure S1A). Rifampicin, an inhibitor of organellar transcription, lengthened the circadian period of *GI:LUC* in DD (Figure 1A and Supplementary Figure S1B) and under BL (Supplementary Figure S1C). The inhibitor of photosynthetic electron transport DCMU lengthened the circadian period of *GI:LUC* under RL and under BL (Figure 1A). However, this effect took place at different concentrations of this chemical depending on the light conditions. Figure 1B shows the period-altering effects of the oxidant paraquat on the period using *GI:LUC* or *CCR2:LUC* [also referred to as *GRP7* (Nicaise et al., 2013)] as reporters of promoter activity under monochromatic light. Under RL, paraquat application shortened the period of *GI:LUC*, whereas it lengthened the period of *CCR2:LUC*. However, under BL, paraquat application lengthened the period of circadian oscillations of both *GI:LUC* and *CCR2:LUC*. Furthermore, the effect was more pronounced and statistically significant with the *CCR2:LUC* marker. As with the effect of DCMU, the plants displayed a higher sensitivity under RL than on BL. Conclusively, the addition to the medium of chemicals known to alter chloroplast-driven metabolic processes affected the circadian-clock parameters.

### Salicylic Acid Action on the Clock

Salicylic Acid signaling has been implicated in connecting environmental stress cues to metabolic reactions driven by the plastid (Muhlenbock et al., 2008; Huang et al., 2010). Moreover, SA is involved in photosynthetic homeostatic regulation in the absence of stress (Rivas-San Vicente and Plasencia, 2011). Hence, SA could be a chemical that links chloroplast function to circadian rhythms. As such, we tested the SA perturbation of clock action in greater depth than in our previous effort (Hanano et al., 2006).

Interestingly, a visual inspection revealed that under RL plus BL, the application of SA increased the robustness of oscillations in all promoter activity reporters tested, which included *GI:LUC* (Figures 1C,E), *CCA1:LUC* (Figures 1D,F), *CCR2:LUC*, and *TOC1:LUC* (Supplementary Figures S1E,F, respectively). To test this further statistically, we distinguished between parameters that define circadian robustness, these being rhythmicity and precision. Here we define rhythmicity as the average of RAE values within a population that represents the fit between the theoretical and the experimental curves after a fast Fourier transform (FFT) analysis has been performed. Precision is defined as the standard deviation of period (descriptive or RAE-normalized, see also “Materials and Methods”). A population of plants generates robust oscillations when individual plants are rhythmic (low RAE values AND high rhythmicity), are in phase with each other, and show similar period values (high precision AND low SD-noPer). Moreover, we distinguish between direct and indirect rhythmicity, the first relating to the mean RAE generated by FFT analysis and the second to the same mean after the plants discarded by the FFT analysis were assigned with an RAE value of 1.

We found that SA application at 1 mM increased the precision and the direct rhythmicity of all reporters tested, whereas SA at 0.5 increased the direct rhythmicity of *TOC1:LUC* and *CCR2:LUC* and increased the precision of *GI:LUC* and *TOC1:LUC* (see Supplementary Tables S6A,B) in a reproducible manner. We should note that the changes in direct rhythmicity mentioned above were minor. Under continuous RL plus BL, the application of SA shortened the circadian period of *GI:LUC*, *CCA1:LUC*, *CCR2:LUC*, and *TOC1:LUC*, but this effect was inconsistent between experiments. Nonetheless, when the results from independent experiments were combined, thus increasing the size of the population, the period shortening effect of SA was statistically significant (see Supplementary Tables S6A,B) for the markers *GI:LUC* (Figure 1C) and *CCA1:LUC* (Figure 1D). This result contradicts our previous report, where SA was not found to have a circadian effect (Hanano et al., 2006). Conclusively, during these early experiments conducted in the presence of supplementary sucrose, the application of SA at high doses affected the circadian parameters and this effect was mostly due to changes in oscillatory precision.

**FIGURE 2:**
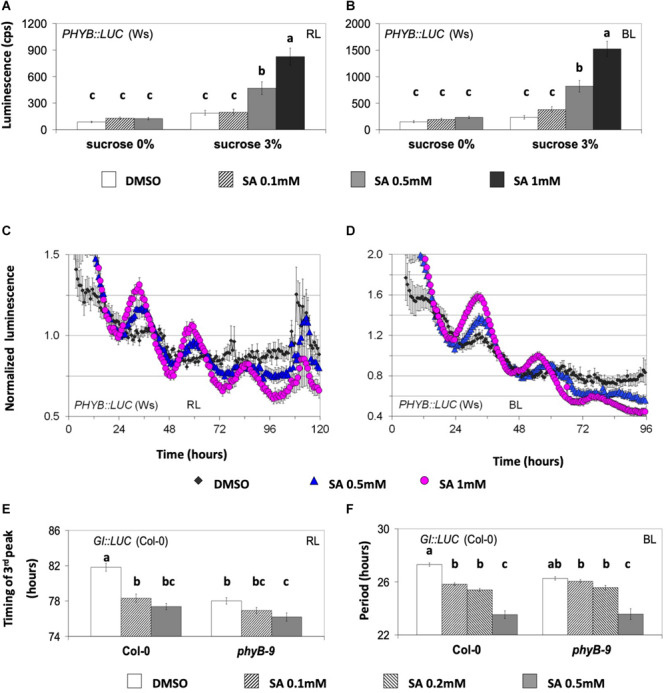
The circadian effect of salicylic acid (SA) is dependent on phyB. **(A,B)** The effect of SA on the expression of *PHYB:LUC* under monochromatic red light (RL, left) and blue light (BL, right) is shown. The plants were entrained on medium with sucrose and then placed on medium with or without sucrose at the indicated SA concentrations. SA at 0.5 mM or higher increased the expression of *PHYB:LUC* only in media supplemented with sucrose. A one-way ANOVA was performed for each dataset (see “Materials and Methods” and Supplementary Tables S7, S8) considering two factors: sucrose concentration and SA concentration. Different letters (a–c) denote statistically significant differences between treatments. The data shown are pooled from several independent assays. Under RL, the bars represent the luminescence of the acute peak that followed dawn. Under BL, the bars represent the luminescence of the first circadian peak. **(C,D)** The effect of SA on the oscillatory robustness of *PHYB:LUC* under monochromatic RL and BL. The experiments were conducted in the presence of supplementary sucrose 3%. SA increased the robustness of *PHYB:LUC* oscillations (see text for details and Supplementary Tables S9A,B). The plants were entrained for one cycle under monochromatic light before being released into free running conditions in the presence of dimethyl sulfoxide (DMSO) or SA, as indicated. **(E,F)** The *phyB* mutant is less sensitive to SA than the wild type. Transgenic plants expressing the *GI:LUC* construct were placed in 96-well microtiter plates containing growth medium without sucrose and either with DMSO or SA. The *phyB-9* mutant was less sensitive than the wild type to SA-mediated phase advance under RL and to SA-mediated period shortening under BL. An ANOVA analysis was performed for each dataset **(E,F)** considering two factors: genotype and SA concentration (see Supplementary Tables S19, S20). Different letters (a–c) denote statistically significant differences between treatments. **(F)** FFT analysis did not include the first circadian peak and spanned at least three cycles. The period interval allowed during FFT analysis was between 15 and 40 h. The gene reporters in **(A–D)** are expressed in the Wassilewskija (Ws) and in **(E)** and **(F)** in the Columbia (Col-0) background. Error bars represent standard error.

We next tested the effect of SA on *PHYB:LUC* expression because phyB is part of SA signaling in defense responses (Genoud et al., 2002). SA application at a concentration of 0.5 mM or more increased the expression under monochromatic RL or BL (Figures 2A,B, Supplementary Tables S7, S8). Interestingly, the inductive effect of SA on the expression of *PHYB:LUC* required sucrose in the medium (Figures 2A,B and Supplementary Tables S6, S7). Under RL plus BL, the expression of *TOC1:LUC* (Supplementary Figure S1G) was increased by SA, while the expression of *GI:LUC* (Supplementary Figure S1H) was decreased. Therefore, we reasoned that the SA-mediated induction of *PHYB:LUC* did not depend on luciferase expression alone since SA changed the expression in a reporter-specific manner. This would exclude the possibility that SA altered the luciferase activity exclusively due to an effect on ATP levels or on the redox state of the cell, although these effects depend on the addition of sucrose to the media.

*PHYB:LUC* was the most responsive marker to SA in terms of oscillatory robustness. Previously, the promoter of *phyB* was shown to be under circadian control (Bognár et al., 1999); however, this oscillation was found to be weak (Toth et al., 2001). We detected that *PHYB:LUC* plants resulted in weak luminescence oscillations that were strengthened in amplitude by SA application in the presence of supplementary sucrose (Figures 2C,D). In more detail, under RL, SA application increased the indirect rhythmicity of the marker at 0.5 and 1 mM and increased its precision at 1 mM; under BL, SA application increased the indirect rhythmicity of the rhythmic marker at 0.5 and 1 mM (statistical analysis shown in Supplementary Tables S9A,B). SA application thus not only increases *PHYB* expression but also increases rhythm robustness.

**FIGURE 3:**
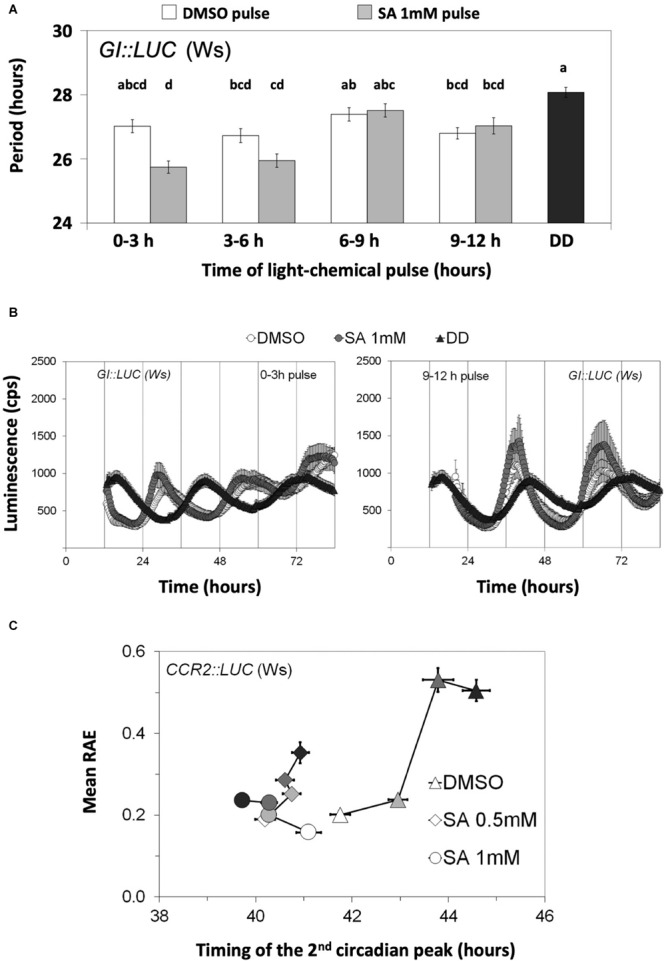
Salicylic acid (SA) affects circadian rhythms through entrainment. **(A)** The effect of SA on the circadian period of *GI:LUC* is gated. Plants were grown and entrained for 5 days under white light and then released into continuous darkness at dusk. A subset of plants was retrieved every 3 h between ZT0 and ZT12 and received a light pulse on medium with 3% sucrose and either dimethyl sulfoxide (DMSO) or SA. Combined data from two independent experiments are shown. The effect of SA on circadian period was gated and restricted to the first 3 h of the day. DD corresponds to the DMSO control that did not receive SA nor light pulses. The results from the ANOVA analysis are shown in Supplementary Table S10. Different letters (a–d) indicate statistically significant differences between means of period. **(B)** Time-course of luminescence obtained from the first and the last chemical/light pulses depicted in **(A)** are shown. **(C)** The effect of SA on the circadian parameters of *CCR2:LUC* is enhanced by parametric entrainment. Populations that did not receive the additional entrainment events are represented by white symbols; pale gray, dark gray, and black symbols correspond to additional entrainment events: 1, 2, or 3, respectively. The plants received the indicated number of entrainment events on medium with sucrose 3% and SA or DMSO in 96-well microtiter plates. Then, they were placed in an automated scintillation counter in continuous darkness and at a constant temperature of 21°C. On the horizontal axis, the medium with DMSO (SA solvent) is shown; consecutive entrainment events in 96-well microtiter plates (0, 1, 2, or 3 days) delayed phase as measured with the timing of the second circadian peak (0 day vs. 1 day, Δphase = 1.20 h, *p* = 3.0 × 10^– 5^; 0 day vs. 2 days, Δphase = 2.03 h, *p* = 4.1 × 10^– 7^; 0 day vs. 3 days, Δphase = 2.82 h, *p* = 4.9 × 10^– 14^). The phase was not substantially affected by such entrainment if SA at 0.5 mM was applied (0.42 h < Δphase < 0.74 h). The application of 1 mM SA reversed the effect of entrainment on phase by the third day (0 days vs. 3 days, Δphase = –1.38 h, *p* = 1.5 × 10^– 6^). Moreover, the SA-mediated phase advances were enhanced by the preceding parametric entrainment events (Supplementary Table S13). On the vertical axis, the SA-mediated increase in indirect rhythmicity is shown to be enhanced by parametric entrainment (Supplementary Table S14). Moreover, consecutive entrainment events in 96-well microplates decreased the indirect rhythmicity and this response was attenuated by SA application. Error bars in all graphs represent standard error.

We then proceeded to test whether SA acts on rhythmic transcription through light and/or entrainment pathways. For this, we subjected the plants to parametric (light/dark cycles) and non-parametric (light pulses given in the dark) entrainment protocols in the presence and the absence of SA (the experiments were conducted in the presence of supplementary sucrose). In non-parametric entrainment experiments, we tested circadian responses to 1 mM SA in a time-course because sensitivity to the hormones ABA, GA, JA, and auxin has been previously reported to be gated by the biological clock (Covington and Harmer, 2007; Legnaioli et al., 2009; Robertson et al., 2009; Arana et al., 2011; Shin et al., 2012). Plants harboring *GI:LUC* were used in the non-parametric entrainment experiments to pulses of light and SA. We found that the effect of SA on circadian period was gated and restricted to the first 3 h of the day (Figure 3A and Supplementary Table S10). Similarly, in these experiments, the effect of SA on phase (timing of the first circadian peak) was greater and statistically significant when pulses were applied between ZT0 and ZT3 relative to later pulses (Figure 3B).

We next examined the effect of continuous SA application on circadian oscillations under parametric entrainment. Plants harboring *CCR2:LUC* (Figure 3C and Supplementary Figure S2A) or *GI:LUC* (Supplementary Figure S2B) were placed on agar with various SA concentrations and entrained under WL for 1, 2, or 3 days. Luminescence rhythms were thereafter monitored in the dark, starting at the last objective dusk. We observed that after 3 days of entrainment had taken place in 96-well microtiter plates, the FFT process did not successfully assign a theoretical curve to 27.56% of *CCR2:LUC*-expressing plants (from a total of 156) and that this percentage dropped to 10.89% by the application of 0.5 mM SA (102 plants) and even to 0% by the application of SA 1 mM (102 plants). In agreement to this, oscillations that produced an FFT output gradually dampened with every entrainment event, unless SA was applied (Supplementary Figure 2A). Similarly, the parametric entrainment of seedlings in 96-well microtiter plates caused the oscillations to be less precise, unless SA was applied (Supplementary Figure 2A and Supplementary Table S11). Consecutive parametric entrainment events in 96-well microtiter plates also caused the oscillations to be less rhythmic, and this effect was attenuated by SA application (Figure 3C and Supplementary Table S12). Moreover, the circadian phase of the control plants was delayed by these consecutive entrainment events; but in the presence of SA, the phase was found to be relatively constant or even advanced with each entrainment event (see Figure 3C for *CCR2:LUC* and Supplementary Figure S2B for *GI:LUC*). Collectively, we found that the entrainment of *Arabidopsis* seedlings in 96-well microtiter plates causes oscillations to dampen and delays the circadian phase, unless SA is applied. In addition to this, a statistical analysis revealed that the effect of SA on the circadian parameters was enhanced by parametric entrainment. This was shown for the combined effects of entrainment and SA application on the indirect rhythmicity of *CCR2:LUC* (Supplementary Table S13) and on the phase of *CCR2:LUC* (Supplementary Table S14) and of *GI:LUC* (Supplementary Table S15). All of these observations were made with as little as 0.2 mM of SA.

**FIGURE 4:**
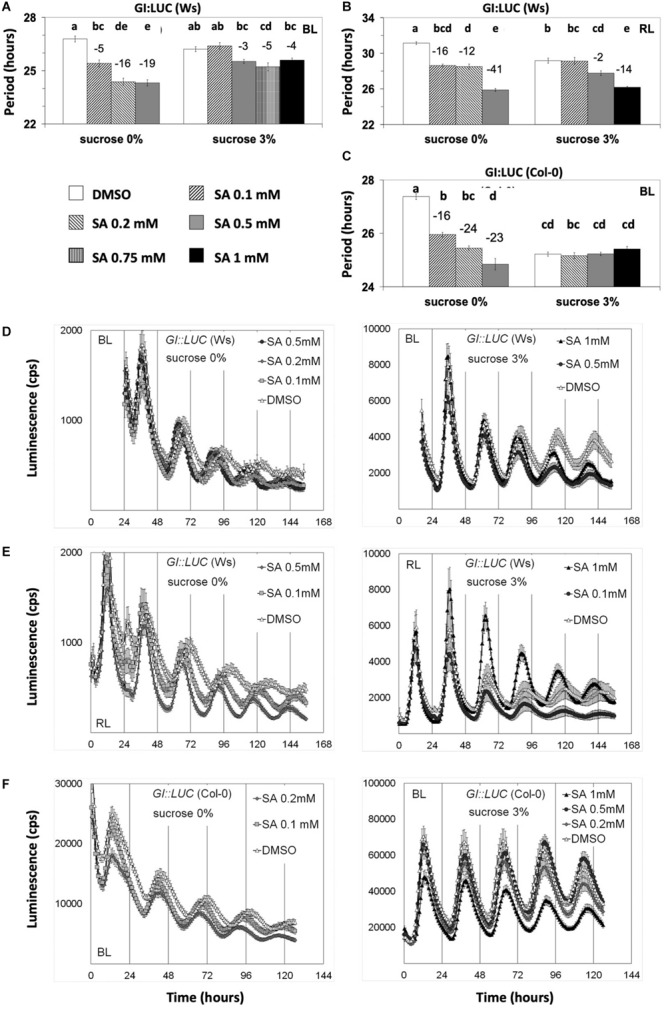
Salicylic acid (SA)-induced period shortening is moderated or inhibited by sucrose. Transgenic plants carrying the designated promoter:luciferase transgenes were grown and entrained under white light before being released into free running conditions under monochromatic blue light (BL) or red light on media with SA and sucrose concentrations as indicated. The period shortening effect of SA was inhibited by sucrose unless a higher concentration of SA was applied. Nonetheless, under BL, even SA at 1 mM could not reduce the period of *GI:LUC*. Reporters in **(A,B,D,E)** and **(F)** are expressed in Wassilewskija, whereas in figures **(C)** and **(F)** in Columbia (Col-0) background. Results from one-way ANOVA statistical analysis for each dataset in **(A–C)** are shown in Supplementary Tables S16–S18, respectively. Different letters (a–e) represent statistically significant differences. Error bars in all graphs represent standard error.

In our assays, the highest SA concentration used (1 mM) caused chlorosis of plants. This could be attributed to SA induction of ROS (Chen et al., 2009), which was our reasoning to include SA in the initial ROS-related chemical screen. We observed that *Arabidopsis* plants were more sensitive to SA-mediated chlorosis if SA was applied without sucrose supplementation. We did not record this, but it is reflected in Figure 4, where SA of 1 mM is applied only when sucrose is supplemented. We should note that this chlorosis observed with SA at 1 mM could not have hindered the luciferase activity as the later was promoter specific (Figures 2A,B, 4D,E and Supplementary Figures S1G,H, S4).

We then proceeded to test if sucrose modifies the effect of SA in circadian assays. We found that, under monochromatic RL and BL, sucrose abolished SA-mediated period shortening of *GI:LUC* (Figure 4 and Supplementary Tables S16–S18) unless this hormone was applied at 0.75–1.0 mM range. Under BL, sucrose prevented the period shortening unless SA was applied with a concentration of 0.75 mM or higher, whereas in media without sucrose, SA at 0.1-mM concentration sufficed to reduce period (Figure 4A). A similar result was observed under RL, with SA requiring concentrations of 0.5 mM in media with sucrose to present period shortening (Figure 4B) and as little as 0.1 mM in media without sucrose. This result was consistent with our previous publication (Hanano et al., 2006) and explains the previous conclusion that SA does not act on the circadian period as Hanano et al. (2006) performed all experiments in the presence of sucrose. It should be noted that non-ionic osmotic stress (Mannitol) at 200 mM lengthens the circadian period (Litthauer et al., 2018), a concentration much higher than the 3% sucrose (∼90 mM) used in this study.

In order to identify loci that mediate SA signaling in the clock, we performed genetic tests with clock mutants. These were *gi-11*, *toc1-21*, *cca1-11*, and *lhy-21.* The *phyB-9* mutant was also tested in this genetic analysis for the distinct response to SA displayed by *PHYB:LUC* (Figure 2) and because the *phyB* mutant is defective in SA signaling during defense responses (Genoud et al., 2002).

*GI:LUC* was used to assess the effect of SA in a phyB context. Under RL, the *phyB-9* (Col-0) mutant was less sensitive to SA-mediated phase advance relative to the wild type (Figure 2E and Supplementary Table S19). Under BL, the phyB mutant similarly was less sensitive to SA-mediated period shortening compared to the wild type (Figure 2F and Supplementary Table S20). In both cases, the mutant required a concentration of 0.5 mM of SA to have an effect. Oscillations in the *phyB-9* mutant were previously reported to be advanced under white light (Salomé et al., 2002), which we confirm under RL here (Figure 2E). Consequently, it cannot be excluded that under RL the early phase phenotype of the *phyB-9* mutant accounts for its decreased sensitivity to SA-mediated phase advance.

**FIGURE 5:**
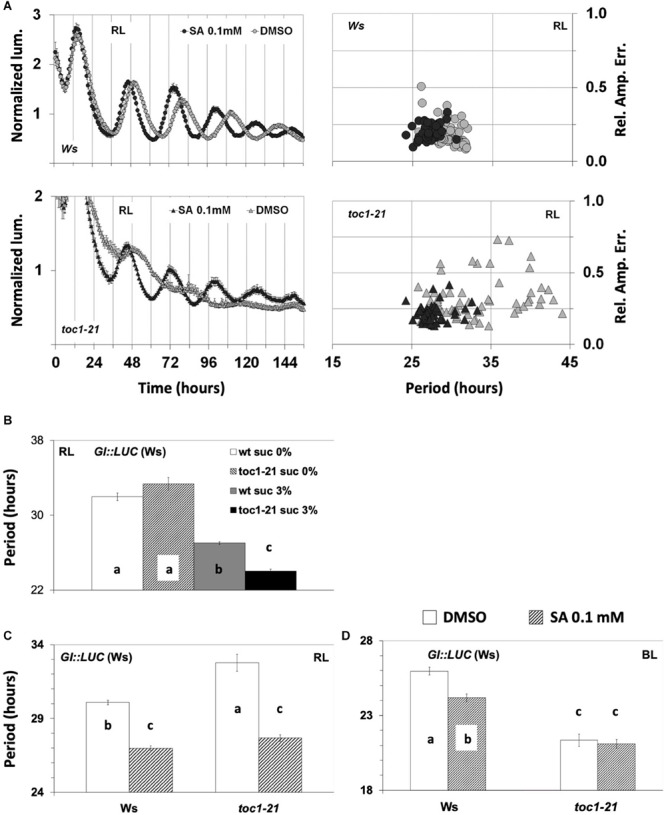
Response of circadian clock mutants to salicylic acid (SA) application. **(A)** The *toc1-21* mutant was more sensitive than the wild type to SA under red light (RL). The experiments were conducted in the absence of supplementary sucrose. In the absence of multiple entrainment events, SA did not improve the oscillatory robustness of *GI:LUC* in the wild type (see also Figure 3C and Supplementary Figure 2A). Note that *toc1-21* treated with SA displays a more concise population, whereas the non-treated is spread, thus has less precision. **(B)** The short period phenotype of the *toc1-21* mutant under RL is sucrose dependent. In the absence of supplementary sucrose, the *toc1-21* mutant exhibited a period similar to that of the wild type; only in the presence of supplementary sucrose did the mutant display a short-period phenotype. Data were analyzed with one-way ANOVA considering two factors: genotype and sucrose concentration (see Supplementary Table S21). **(C)** SA application under RL shortened the circadian period of both the wild type and the *toc1-21* mutant in media without sucrose. Note that without sucrose the mutant period length is longer than that of the wild type. The output of the ANOVA analysis with two factors is shown in Supplementary Table S22. **(D)** The *toc1-21* mutant is irresponsive to period shortening by SA application under blue light in media without sucrose. Note that under these conditions the mutant displayed a short period. Results of the data analysis are shown in Supplementary Table S23. In **(B–D)** different letters (a–c) denote statistically significant differences between means of period means. Error bars in all graphs represent standard error.

We analyzed the effect of SA application on the *toc1-21* mutant under monochromatic RL or BL in the absence of sucrose. *GI:LUC* was used to assess the rhythm. Previously, it was shown that TOC1 is required for oscillations of *CCR2:LUC* and *CAB2:LUC* under monochromatic RL in experiments where sucrose was supplemented (Más et al., 2003a). Here we show that the *GI:LUC* construct exhibits weak oscillations in the *toc1-21* background that were strengthened by SA application (Figure 5A). The FFT analysis yielded a free running period for the mutant that was, strikingly, slightly longer than that of the wild type (Figures 5A,C). *toc1-21* is known to be a short period mutant (Strayer et al., 2000; Alabadí et al., 2001), and because this phenotype has been reported in the presence of sucrose, we proceeded to test whether the *toc1-21* phenotype under RL is sucrose dependent. Figure 5B shows that, under monochromatic RL, the short period phenotype of the *toc1-21* mutant is sucrose dependent (see also Supplementary Table S21). The *toc1-21* mutant did not display a short period phenotype in the absence of sucrose, even when the plants were placed on agar with 0.1 mM of SA that restores the oscillations in the mutant (compare the dashed bars in Figure 5C and the black curve in Figure 5A; the period values are shown in Supplementary Tables S21, S22). It is noteworthy that, under red light (Figure 5C), the *toc1-21* mutant exhibited a long period phenotype, whereas under BL we recorded a short period phenotype of the *toc1-21* mutant (Figure 5D and Supplementary Table S23), which was not affected by SA application. Previously, we have shown that the *lhy-21*, *cca1-11*, and *gi-11* mutants also show sucrose-dependent phenotypes (Philippou et al., 2019).

The *toc1-21* mutant was oversensitive to SA under RL. As expected, in the absence of sucrose, the wild-type plants responded to SA with period shortening, while the mutant responded similarly but to a greater extent (Figures 5A,C and Supplementary Tables S21, S22). The oversensitivity phenotype of *toc1-21* to SA was also seen for the effect of SA on oscillatory robustness, which was mostly due to changes in precision (see how the black dots collide together compared to the more dispersed gray dots in Figure 5A, lower panel). Under BL, the *toc1-21* mutant was less sensitive to SA-mediated period shortening than the wild type (Figure 5D and Supplementary Table S23). This was observed in experiments conducted without sucrose supplementation, either with the *GI:LUC* construct (Figure 5D and Supplementary Table S23) and at least in one experiment with the *CAB2:LUC* construct (Supplementary Figure S3). As such, light quality had a significant impact on the SA-related circadian phenotypes of *toc1-21*, the mutant being oversensitive to SA under RL and less sensitive under BL than the wild type. However, it cannot be excluded that under BL the short period phenotype of *toc1-21* accounts for its reduced sensitivity to SA-mediated period shortening.

**FIGURE 6:**
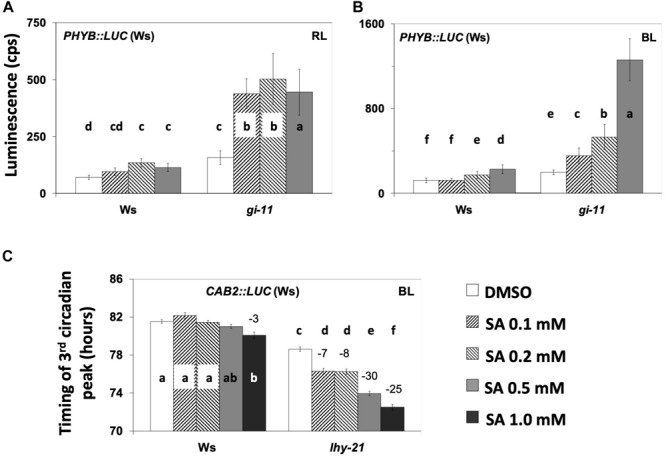
**(A,B)** The *gi-11* mutant has an exacerbated increase in the expression of *PHY:LUC* by salicylic acid (SA) application under red light (RL) **(A)** and blue light (BL) **(B)** on medium without sucrose. SA increased the expression of marker *PHYB:LUC*, although this response was exacerbated in the *gi-11* mutant requiring solely 0.1 mM of SA to produce this effect compared to a higher SA concentration in the wild type. Under RL, bars represent the luminescence of the acute peak that followed dawn; under BL, bars represent the luminescence of the first circadian peak that followed the acute peak of dawn. Statistical analysis are shown in Supplementary Tables S24, S25. **(C)** SA application diminishes the expression of *CAB2:LUC* in the *lhy-21* mutant under BL in the presence of supplementary sucrose. Note that under these conditions, the wild type did not respond to SA application even at 1.0 mM, whereas the *lhy-21* mutant was hypersensitive. ANOVA statistical results are shown in Supplementary Table S26. Different letters (a–f) denote statistically significant differences between treatments. Error bars in all graphs represent standard error.

It has been suggested that GI acts within light-input pathways (Park et al., 1999; Locke et al., 2006) *via* phyB signaling in particular (Huq et al., 2000). Moreover, phyB is recognized as a mediator of SA signaling during defense responses (Genoud et al., 2002). Thus, we proceeded to examine whether the effect of SA on *PHYB:LUC* expression (observed in Figures 2A,B) is modified in the *gi-11* background. We found that the *gi-11* mutant was consistently oversensitive to SA relative to the wild type when analyzing the effect of the hormone on the expression of *PHYB:LUC* either under RL or BL (Figures 6A,B and Supplementary Tables S24, S25). It is worth noting that this oversensitivity phenotype was observed on the medium that did not contain sucrose.

We next tested the *lhy-21* (Figure 6C and Supplementary Table S26) and *cca1-11* (Supplementary Figure S4) mutants for their responses to SA under BL with *CAB2:LUC*. Luminescence rhythms indicated that, in the presence of supplementary sucrose, *lhy-21* was responsive to SA with period shortening, unlike the wild type. A FFT–non-linear least squares (NLLS) analysis confirmed this in only two out of the four experiments conducted. Consequently, we calculated the timing of the third peak after release into free running conditions and found that *lhy-21* was more sensitive to SA-mediated peak advance than the wild type in every experiment (Figure 6C and Supplementary Figure S4, Supplementary Tables S26, S27). *cca1-11* did not display a detectable SA-related phenotype in terms of period or phase (timing of the third peak of oscillations) (Supplementary Figure S4). Thus, here we only detected that the *lhy* mutant displayed a SA-mediated phenotype.

## Discussion

### Chemical Perturbation of Chloroplast Function Is Reflected in Nuclear Oscillations

The pace of the clock is resilient to most chemicals as the application of thousands of compounds of various structures has no action on clock performance (Toth et al., 2012). Interestingly, the chemicals that we examined alter the circadian parameters and are related to chloroplast function (Figure 1). Thus, our data support the notion that photosynthesis and ETCs exert an input to nuclear oscillations.

Rifampicin, an inhibitor of organellar transcription, lengthened the circadian period in the dark as well as under continuous light. Previously, Vanden Driessche et al. (1970) and Mergenhagen and Schweiger (1975) reported that rifampicin does not affect the rhythmic oxygen evolution from individual cells of the unicellular algae *Acetabularia*. The antioxidant vitC and the oxidant paraquat altered the circadian period in a light quality- and reporter-specific manner. The importance of vitC in photosynthesis is underlined by its high concentration in chloroplasts (20–300 mM). Its photo-protective activities are manifested in the regulation of the redox state of photosynthetic electron carriers in the direct or the enzymatic detoxification of ROS and in the role of vitC as an enzymatic cofactor during thermal dissipation of excess excitation energy (Smirnoff, 2000). Paraquat is a non-selective contact herbicide that generates ROS by accepting electrons from photosystem I (PSI) and transfers them to molecular oxygen. Interestingly, the *gi* mutant was shown to be resistant to paraquat-induced oxidative stress (Kurepa et al., 1998), whereas the circadian clock-related mutant *time for coffee* (*tic-2*) is overly sensitive to it (Sánchez-Villarreal et al., 2013), although it is not known if this behavior is related to a circadian phenotype (Shin et al., 2013). However, Lai et al. (2012) demonstrated that CCA1 acts as a master regulator of oxidative stress within the circadian clock. DCMU, which lengthened the circadian period in our experiments, is known to shift the PQ poll to its oxidized state as it inhibits the photosynthetic ETC upstream of PQ. The relationship of SA to chloroplasts has been reported in several studies (see the references below). Altogether our data are consistent with the hypothesis that chloroplast energy homeostasis creates a plastid-derived signal that intersects with the nuclear TTO to define a circadian period.

Major effects on entrainment were found to be altered by SA application (Figure 3 and Supplementary Figure S2). This could relate to the nature of the hormone. SA is increased after exposure to high light (Chang et al., 2009) and contributes to acclimation and photosynthetic energy dissipation through photorespiration (Mateo et al., 2004) as well as through the induction of the antioxidant molecule glutathione (Mateo et al., 2006) and likely vitC (Chang et al., 2009). Thus, it makes sense to observe an effect early in the daytime (Figures 3A,B) as previously found by Covington et al. (2008).

A role for phyB in red light and blue light input to the clock (in the absence of supplementary sucrose) is supported by the reduced sensitivity of the *phyB-9* mutant to SA (Figures 2E,F). It is noteworthy that phyB is required downstream of SA signaling during certain aspects of host-plant defense mechanisms, such as the hypersensitive response that requires functional chloroplasts (Genoud et al., 2002). Our work provides further evidence that a pathway involving SA functions during parametric and non-parametric light entrainment (Figure 3). These findings together raise the possibility that the aforementioned pathway involved in defense responses, might also relate to photic entrainment. It is noteworthy that photic entrainment in cyanobacteria does not require photoreceptors. In this case, light input to the clock and circadian entrainment have been connected to the redox state of the photosynthetic ETC and the redox state of the PQ pool (Mackey et al., 2011).

### ETCs Affect Nuclear Oscillations

The role of ETCs in the regulation of a given process has been shown with distinct experimentation. Yabuta et al. (2007) have suggested that vitC levels are under the regulation of photosynthetic ETCs rather than of sugars because DCMU and sucrose both had a negative impact on the accumulation of vitC after exposure to continuous light. This argument was based on the fact that, similarly to DCMU, photosynthates inhibit photosynthesis (Paul and Foyer, 2001). The involvement of ETCs in the regulation of a given process has also been demonstrated through the controlled manipulation of the redox state of PQ by chemicals and light quality. In more detail, treatment of low light-grown plants with the inhibitors of photosynthetic ETCs DCMU or DBMIB elicits similar effects on the redox status of the PQ pool as light enriched with far red light (FRL, 700 nm) or red light (RL, 680 nm). DCMU and FRL cause the oxidation of PQ, while DBMIB and RL cause the reduction of PQ. An antagonistic effect between these factors is therefore indicative that a process is sensitive to signals derived from PQ (Pfannschmidt et al., 2009). We found that the photosynthesis inhibitor DCMU and SA at low concentrations, which favors photosynthetic electron transport (Rivas-San Vicente and Plasencia, 2011), had opposite effects on circadian period. This would suggest that nuclear oscillations are under the regulation of ETCs. Our observation that the period-shortening effect of SA was inhibited by sucrose (see Figure 4 and Supplementary Figure S4) further supports this notion as photosynthates, including sucrose, inhibit photosynthetic activity (Koch, 1996). Moreover, our results suggest that under BL, DBMIB (Supplementary Figure S5) and DCMU (Figure 1A) did not perturb the clock synergistically. Consequently, DCMU might lengthen the circadian period through its effect on the redox state of the PQ pool.

### Photosynthetic Electron Transport Activity Might Be Correlated to Circadian Period

Wenden et al. (2011) showed that, under RL, the circadian period is shorter than under FRL. This observation and the results presented in Figure 1 suggest a correlation between photosynthetic electron transport activity and circadian period. Factors that reduce the PQ pool, such as RL, and those that could exert a protective role during photosynthesis through the regulations of PSII, such as SA and vitC (Karpinski et al., 1999; Smirnoff, 2000), induce period shortening, whereas factors that cause oxidation of the PQ pool such as DCMU, FRL (Muhlenbock et al., 2008), DD, or low light intensity (Oswald et al., 2001), or that inhibit photosynthesis, such as rifampicin (Figure 1) and iron deficiency [reviewed in Wilson and Connolly (2013)], all promote period lengthening. This correlation between the expected changes in the redox state of the PQ pool and the observed changes in the circadian period was also seen with the oxidant paraquat under blue light (Figure 1B). This is further supported by the aforementioned experiments with DBMIB and DCMU in which these photosynthesis inhibitors did not affect the circadian period similarly (compare Figure 1A with Supplementary Figure S5).

This and the reported studies together suggest a positive correlation between circadian period length and electron transport downstream of PSII. Based on this correlation, we propose the following: (a) ETCs might be involved in photic entrainment. This is further implied by the fact that the circadian effect of SA, directly connected to entrainment (Figure 3), is inhibited by sucrose application (Figure 4) that also inhibits photosynthesis (Koch, 1996); (b) ambient light intensity would contribute to circadian period, as predicted by the rule of Aschoff and FRCs, through the observed effect of fluence rate on the redox state of PQ (Oswald et al., 2001). In agreement, James et al. (2008) showed that the root clock, lacking photosynthetic activity, does not obey the rule of Aschoff; (c) oscillations in SA levels (Goodspeed et al., 2012) and in SA time-specific activity (Figure 3) and potential oscillations in photosynthetic electron transport would meet certain criteria as predicted by the *zeitnehmer* model (see below).

Mathematical modeling (Roenneberg and Merrow, 1999), confirmed experimentally in *Neurospora* (Merrow et al., 1999), has led to the identification of certain criteria that define *zeitnehmer* loops. Amongst these criteria are (1) rhythmicity *per se* of a biochemical pathway, the *zeitnehmer*, that perceives *zeitgeber* signals for the purpose of entrainment and then (2) through coupling of the *zeitnehmer* loop to a central oscillator provision of rhythm sustainability. The gated effect of SA on circadian timing (Figure 3A) implies an oscillatory potential in SA signaling. This is also suggested by the observation that SA levels are circadian-regulated (Goodspeed et al., 2012). Moreover, SA was shown here to be involved in parametric and non-parametric entrainment (Figure 3 and Supplementary Figure S2) as well as in rhythm sustainability (Figures 2C,D, 3C, 5A and Supplementary Figure S2). This strongly supports that SA is directly or indirectly involved in a *zeitnehmer* loop that could entrain nuclear oscillations and provide rhythm sustainability. Coupling between TTOs and SA signaling or a related process is further supported by the SA-related phenotypes of *phyB-9* (Figure 2), *toc1-21* (Figure 5), *gi-11*, and *lhy-21* (Figure 6). Photosynthetic electron transport might be a potential candidate for such an SA-related process, given the correlation between the expected changes in the redox state of the PQ pool and the observed changes in circadian period presented here and in the literature. It is noteworthy that retrograde signaling and ROS produced as a consequence of the normal functioning of photosynthesis and respiration are being considered in the literature as circadian determinants (Dodd et al., 2015; Guadagno et al., 2018; Jones, 2018).

## Materials and Methods

### Plant Materials

Rhythmicity was monitored using the promoter:luciferase system (Gould et al., 2006; Hanano et al., 2006; Kevei et al., 2006) in the *A. thaliana* Columbia (Col-0) and Wassilewskija (Ws) genetic backgrounds. Rhythmic promoter:luciferase markers in the Ws wild-type background are described in the literature as follows: *CCR2:LUC*, *CCA1:LUC* (Doyle et al., 2002), *CAB2:LUC* (Hall et al., 2001), *TOC1:LUC* (McWatters et al., 2007), *GI:LUC* (Ding et al., 2007), *PHYB:LUC* (Toth et al., 2001), *cca1-11*, *lhy-21*, and *toc1-21* mutants with the *CAB2:LUC* marker (Ding et al., 2007), and *CAB2:LUC* in the *gi-11* mutant (Gould et al., 2006); *PHYB:LUC* was introduced in the *gi-11* mutant and *GI:LUC* in the *toc1-21* by crossing transgenic plants expressing *GI:LUC* in the wild-type Col-0 background and in the *phyB-9* mutant (Oh et al., 2004).

### Growth Conditions and Luciferase Imaging

The seeds were surface-sterilized, sown on 1% agar containing Murashige and Skoog basal salt mixture (pH 5.7) (Murashige and Skoog, 1962), and stratified for 3 days. The seedlings were entrained under 12-h light/12-h dark photoperiods under a fluence rate of white light (WL) at 100 μmol m^–2^ s^–1^ and a constant temperature of 22°C. During the second half of the subjective day and before dusk, the 6-day-old seedlings were transferred into 96-well microtiter plates (Perkin Elmer, Jügesheim, Germany) containing agar with chemicals or their respective diluents [dimethyl sulfoxide (DMSO) or water in the case of vitC] as controls with or without sucrose (3% w/v) as indicated. The seedlings were imaged in a luminescence scintillation counter (TopCount NXT, Perkin Elmer) at dusk (Southern and Millar, 2005; Hanano et al., 2006), allowing imaging under low fluence rates of red light (RL) and blue light (BL). The plants received a dark period of 12 h that corresponds to the subjective night and then entered free running conditions under monochromatic RL or BL at a low fluence rate (∼2 μmol m^–2^ s^–1^) provided by LEDs (Boikoglou et al., 2011). In some experiments, an additional entrainment event was applied in the automated scintillation counter before the onset of the free run.

### Data Analysis

The luminescence levels were quantified and graphically depicted using TopTempII and Biological Rhythms Analysis Software System (Southern and Millar, 2005). Period length and RAE were estimated using the FFT–NLLS program (Plautz et al., 1997).

To assess differences in period between and within chemical treatments, genotypes, and/or lighting conditions, we performed a one-way ANOVA with *post hoc* Tukey for multiple testing, using SAS 9.0 with default parameters (*p*-value 0.05). We used a two-factor experimental design for most of the data (Figures 1A,B, 2F, 4A–C, 5B–D, 6A–C) or a completely random design (Supplementary Tables S3, S10). Each analyzed dataset results are depicted in the supplementary tables and in Figure 1A separated within the figure with dashed lines. Statistically significant differences are shown with different letters in each panel and/or figure. Similarly, to evaluate differences in luminescence and the timing of the third peak (Figures 2A,B,E, 6), we used a one-way ANOVA with Tukey for multiple testing, using SAS 9.0 with default parameters (*p*-value 0.05) using a two-factor experimental design. For simpler datasets, Student’s *t*-test was used to compare between two populations using Microsoft Excel (see Supplementary Tables).

Period length is either a descriptive (not normalized) or a RAE-normalized period (noPer), in which case the contribution of a given period measurement is negatively correlated to its corresponding RAE value. The *p* values for differences in period (or any other circadian parameter) refer to descriptive data. Therefore, both measures for period are presented, RAE-normalized in graphs and with *p* values after a Student’s *t*-test. The precision of a rhythmic population is defined by its inverse relation to the standard deviation (SD) of period (normalized or descriptive). The rhythmicity of a rhythmic population is defined by its inverse relation to the SD of period (normalized or descriptive). We distinguish between direct and indirect rhythmicity, the first relating to the mean RAE generated by FFT analysis and the second to the same mean after the plants discarded by FFT analysis were assigned with a RAE value of 1.

Sinusoidal curves represent luminescence activity or luminescence normalized to luciferase activity. Luminescence was automatically averaged by TopTempII for each plant separately. The normalized luminescence graphs were then generated by TopTempII for each population (Figures 2C,D, 5A).

To quantify the expression of the luminescence of *PHYB:LUC* (Ws), the timing of the first acute peak after dawn (Figure 2A) or the first circadian peak (Figure 2B) was defined for each oscillating population by a visual inspection of the normalized luminescence graphs generated in TopTempII. The average luminescence at that time point was then used to assess the effect of SA on the expression of the marker.

### Light/Chemical Pulse Experiments

The range of the chemicals tested is illustrated in Table 1. For the experiments presented in Figures 3A,B, the plants harboring *GI:LUC* construct were entrained for 5 days under 12-h light/12-h dark photoperiod at a constant temperature of 22°C before entering continuous darkness at dusk. This was similar to the carbon and sucrose examinations by Perea-García et al. (2015); Perea-Garcia et al. (2016), and Philippou et al. (2019). Briefly, every 3 h, a subset of plants was retrieved from the basal growth medium (MS) and subjected to non-parametric entrainment with 3-h light pulses (WL) on the growth medium that contained SA at 1 mM or DMSO. The chemical pulse was 15 min shorter than the light pulse for technical reasons. At the end of each light/chemical pulse, the plants were placed in 96-well microtiter plates on basal MS media (without SA or DMSO) and luminescence was monitored in continuous darkness. Four time windows for light–chemical pulses were applied between ZT0 and ZT12 hours (ZT for *zeitgeber* time; ZT0 is objective dawn that marks the beginning of the free run).

**TABLE 1:** Range of concentrations reported in the literature for the chemicals that affected the circadian period in this study.

Chemical	Active concentration in the present study	Concentration used in the cited reference	References
SA	0.1–1 mM	0.25–0.5 mM	Genoud et al. (2002)
DCMU	5–10 μM	8 μM	Muhlenbock et al. (2008)
DBMIB	5–10 μM	14 μM	Muhlenbock et al. (2008)
VitC	2–3 mM	10 mM	Horling et al. (2003)

*Rifampicin concentrations vary widely in the literature.*

## Data Availability Statement

All datasets generated for this study are included in the article/Supplementary Material.

## Author Contributions

All authors contributed to the writing of this manuscript. KP, AS-V, and AD contributed to the work. SD oversaw the study.

## Conflict of Interest

The authors declare that the research was conducted in the absence of any commercial or financial relationships that could be construed as a potential conflict of interest.

## Supplementary Material

FIGURE S1 Effect of different chemicals on circadian rhythm. **(A)** Vitamin C did not alter the circadian period of *GI:LUC* under blue light (BL). Standard errors were omitted for clarity. **(B,C)** Rifampicin lengthened the circadian period of *GI:LUC* in the dark **(B)** and under BL **(C)**. **(D)** Salicylic acid (SA) induces a short period of *CCR2:LUC* under BL. **(E,F)** SA increased the oscillatory robustness of *CCR2:LUC* **(E)** and of *TOC1:LUC* **(F)**; horizontal error bars represent the standard deviation (SD) of period and vertical error bars represent the standard error of the relative amplitude error. Note that SA application diminishes the SD. **(G,H)** Effect of SA on luciferase activity under RL plus BL is marker-specific. SA increased the expression of *TOC:LUC* **(G)** and decreased the expression of *GI:LUC* **(H)**. Consequently, the effect of SA on luciferase activity, being marker-specific, could not be dependent on luciferase activity alone. Experiments were conducted in the presence of supplementary sucrose. Growth and entrainment took place as described in Figure 1. Error bars represent standard errors in **(A–D,G,H)**.

FIGURE S2 Salicylic acid (SA) affects the circadian rhythms through entrainment. **(A)** SA increases the oscillatory robustness of *CCR2:LUC* through parametric entrainment. The plants received the indicated number of entrainment events on medium with 3% sucrose and SA or dimethyl sulfoxide (DMSO) in 96-well microtiter plates. Then, the seedlings were placed in a TopCount in continuous darkness and at a constant temperature of 21°C. The panels on the right represent the combined data from three independent experiments that produced similar results to each other. The panels on the left show one of these experiments; the error bars represent standard error and are occasionally smaller than the respective symbols. The *y*-axis in the right panels is negatively correlated to direct rhythmicity and the standard deviation on the *x*-axis is negatively correlated to precision. **(B)** Consecutive entrainment events (0, 1, 2, or 3 days and in one experiment 7 days) in 96-well microplates delayed the phase (timing of the second circadian peak) of *GI:LUC* expression (0 day vs. 1 day, Δphase = 1.33 h, *p* = 0.01; 0 day vs. 2 days, Δphase = 3.49 h, *p* = 1.3 × 10^–4^; 0 day vs. 3 days, Δphase = 4.29 h, *p* = 6.0 × 10^–13^; 0 day vs. 7 days, Δphase = 3.13 h, *p* = 8.8 × 10^–4^). Phase, however, did not change after 7 days of entrainment if SA was applied at 0.1 mM (Δphase = 1.18 h, *p* = 0.10). Application of SA at 0.5 mM reversed the effect of entrainment on phase by the 7th day (0 day vs. 7 days, Δphase = −1.84 h, *p* = 3.1 × 10^–4^). Similarly, the application of SA at 1 mM resulted in phase advances (0 d vs. 1 day, Δphase = −2.69 h, *p* = 2.9 × 10^–5^; 0 day vs. 2 days, Δphase = −1.18 h, *p* = 0.01; 0 day vs. 3 days, Δphase = −0.70 h, *p* > 0.05). Student’s *t*-test for each pair comparison is shown. Statistical analysis showed that the SA-mediated phase advances were enhanced by the preceding parametric entrainment events. Error bars in all graphs represent standard error.

FIGURE S3 Salicylic acid (SA)-related phenotypes of the *toc1-21* mutant. Under blue light, in the absence of supplementary sucrose, the application of SA at 0.1 mM accelerated the oscillations of *CAB2:LUC* in the wild-type (*p* = 0.01) but not in the *toc1-21* mutant (*p* = 0.79). Error bars represent standard error.

FIGURE S4 **(A,B)** Sucrose moderated the period-shortening effect of salicylic acid (SA) of the wild type harboring the *CAB2:LUC* transgene. **(A,C)** The *cca1-11* mutant did not display any SA-related circadian phenotypes. **(B,D)** The *lhy-21* mutant was slightly more sensitive than the wild type to SA-mediated peak advance [third peak shown with arrow in **(D)**]. Note that **(D)** does not display the first two peaks from *zeitgeber* time 0–48 h as in the other figures. Error bars represent standard error.

FIGURE S5 2,5-Dibromo-3-methyl-6-isopropylbenzoquinone, unlike 3-(3,4-dichlorophenyl)-1,1-dimethylurea (Figure 1A), did not disturb the rhythmic expression of *GI:LUC* under blue light. The error bars are smaller than the symbols and represent standard error.
