# Supplementary figures and images for: Chemical Perturbation of Chloroplast-Related Processes Affects Circadian Rhythms of Gene Expression in Arabidopsis: Salicylic Acid Application Can Entrain the Clock
Source: Front Physiol. 2020 Jun 18;11:429. doi: 10.3389/fphys.2020.00429 (PMC7314985; doi:10.3389/fphys.2020.00429)

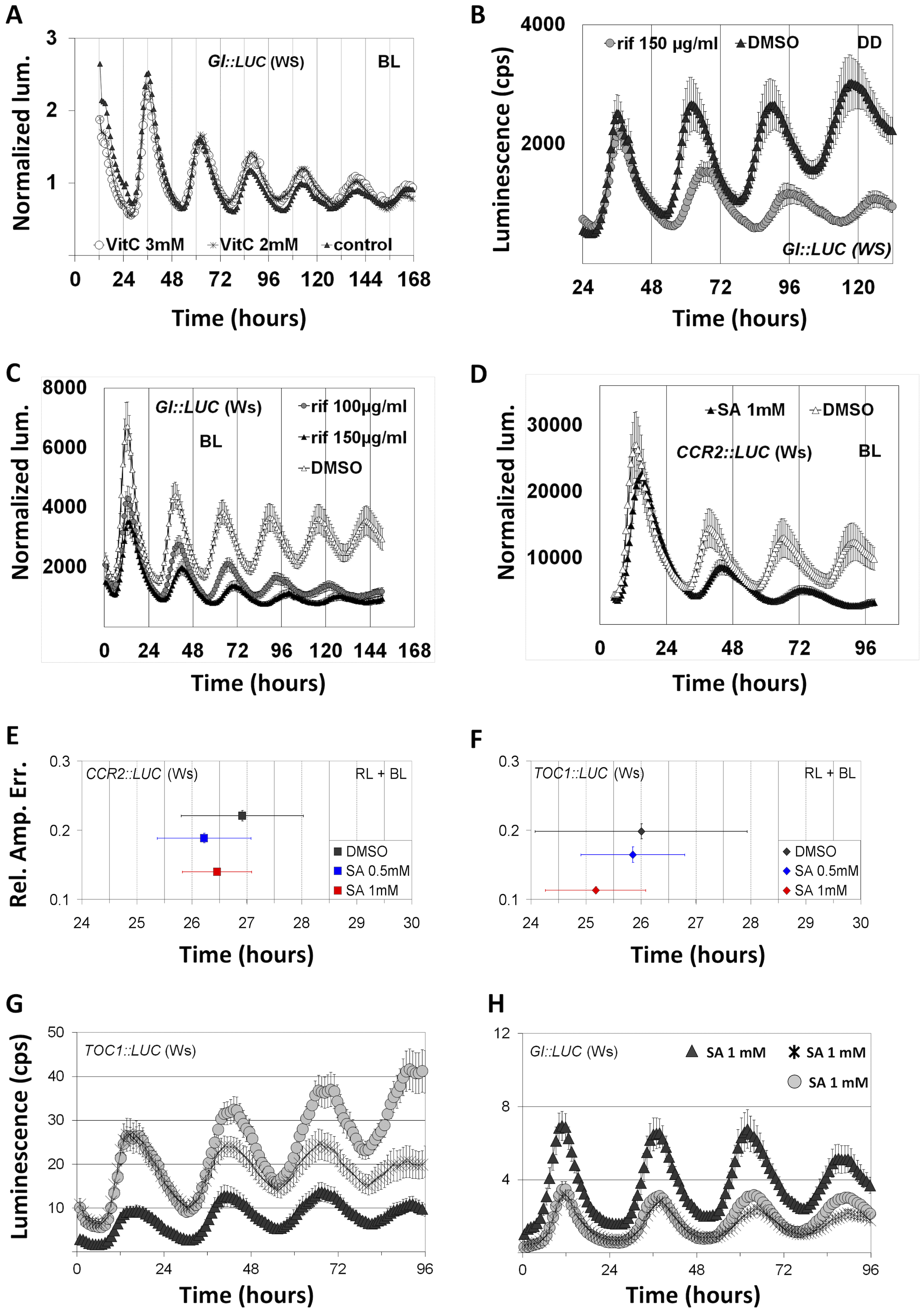

Supplement: FIGURE S1 — Effect of different chemicals on circadian rhythm. (A) Vitamin C did not alter the circadian period of GI:LUC under blue light (BL). Standard errors were omitted for clarity. (B,C) Rifampicin lengthened the circadian period of GI:LUC in the dark (B) and under BL (C). (D) Salicylic acid (SA) induces a short period of CCR2:LUC under BL. (E,F) SA increased the oscillatory robustness of CCR2:LUC (E) and of TOC1:LUC (F); horizontal error bars represent the standard deviation (SD) of period and vertical error bars represent the standard error of the relative amplitude error. Note that SA application diminishes the SD. (G,H) Effect of SA on luciferase activity under RL plus BL is marker-specific. SA increased the expression of TOC:LUC (G) and decreased the expression of GI:LUC (H). Consequently, the effect of SA on luciferase activity, being marker-specific, could not be dependent on luciferase activity alone. Experiments were conducted in the presence of supplementary sucrose. Growth and entrainment took place as described in Figure 1. Error bars represent standard errors in (A–D,G,H). [file Image_1.jpg]

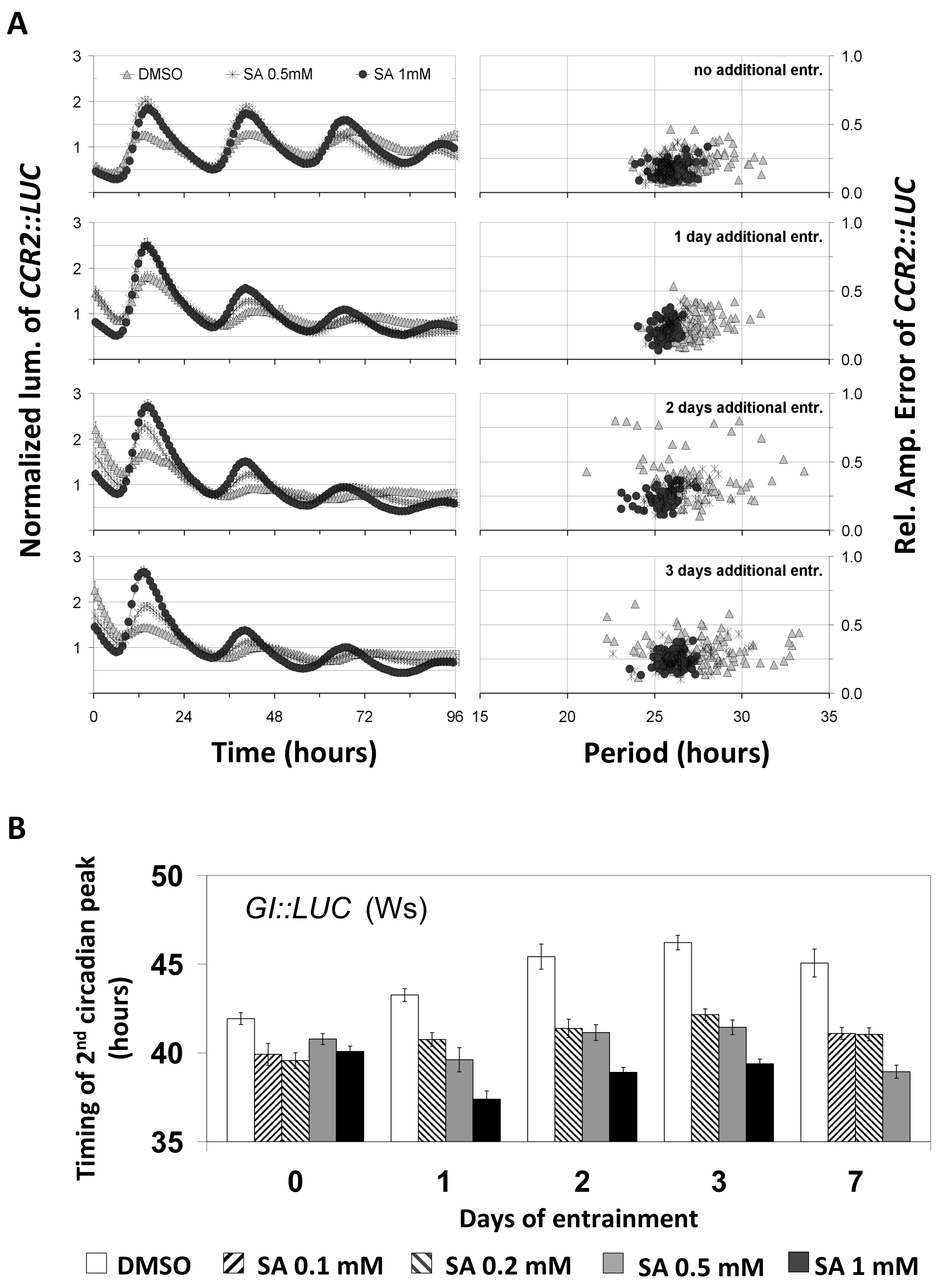

Supplement: FIGURE S2 — Salicylic acid (SA) affects the circadian rhythms through entrainment. (A) SA increases the oscillatory robustness of CCR2:LUC through parametric entrainment. The plants received the indicated number of entrainment events on medium with 3% sucrose and SA or dimethyl sulfoxide (DMSO) in 96-well microtiter plates. Then, the seedlings were placed in a TopCount in continuous darkness and at a constant temperature of 21°C. The panels on the right represent the combined data from three independent experiments that produced similar results to each other. The panels on the left show one of these experiments; the error bars represent standard error and are occasionally smaller than the respective symbols. The y-axis in the right panels is negatively correlated to direct rhythmicity and the standard deviation on the x-axis is negatively correlated to precision. (B) Consecutive entrainment events (0, 1, 2, or 3 days and in one experiment 7 days) in 96-well microplates delayed the phase (timing of the second circadian peak) of GI:LUC expression (0 day vs. 1 day, Δphase = 1.33 h, p = 0.01; 0 day vs. 2 days, Δphase = 3.49 h, p = 1.3 × 10^–4^; 0 day vs. 3 days, Δphase = 4.29 h, p = 6.0 × 10^–13^; 0 day vs. 7 days, Δphase = 3.13 h, p = 8.8 × 10^–4^). Phase, however, did not change after 7 days of entrainment if SA was applied at 0.1 mM (Δphase = 1.18 h, p = 0.10). Application of SA at 0.5 mM reversed the effect of entrainment on phase by the 7th day (0 day vs. 7 days, Δphase = −1.84 h, p = 3.1 × 10^–4^). Similarly, the application of SA at 1 mM resulted in phase advances (0 d vs. 1 day, Δphase = −2.69 h, p = 2.9 × 10^–5^; 0 day vs. 2 days, Δphase = −1.18 h, p = 0.01; 0 day vs. 3 days, Δphase = −0.70 h, p > 0.05). Student’s t-test for each pair comparison is shown. Statistical analysis showed that the SA-mediated phase advances were enhanced by the preceding parametric entrainment events. Error bars in all graphs represent standard error. [file Image_2.jpg]

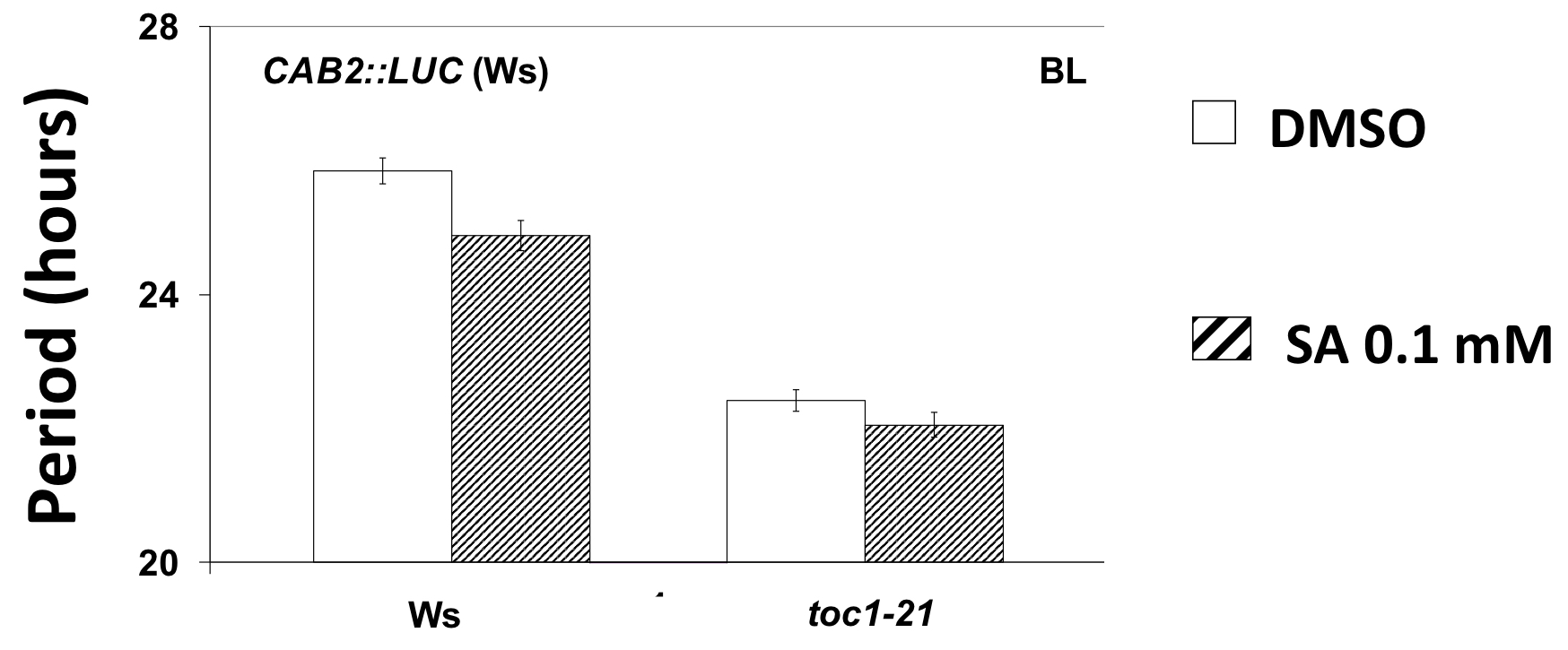

Supplement: FIGURE S3 — Salicylic acid (SA)-related phenotypes of the toc1-21 mutant. Under blue light, in the absence of supplementary sucrose, the application of SA at 0.1 mM accelerated the oscillations of CAB2:LUC in the wild-type (p = 0.01) but not in the toc1-21 mutant (p = 0.79). Error bars represent standard error. [file Image_3.jpg]

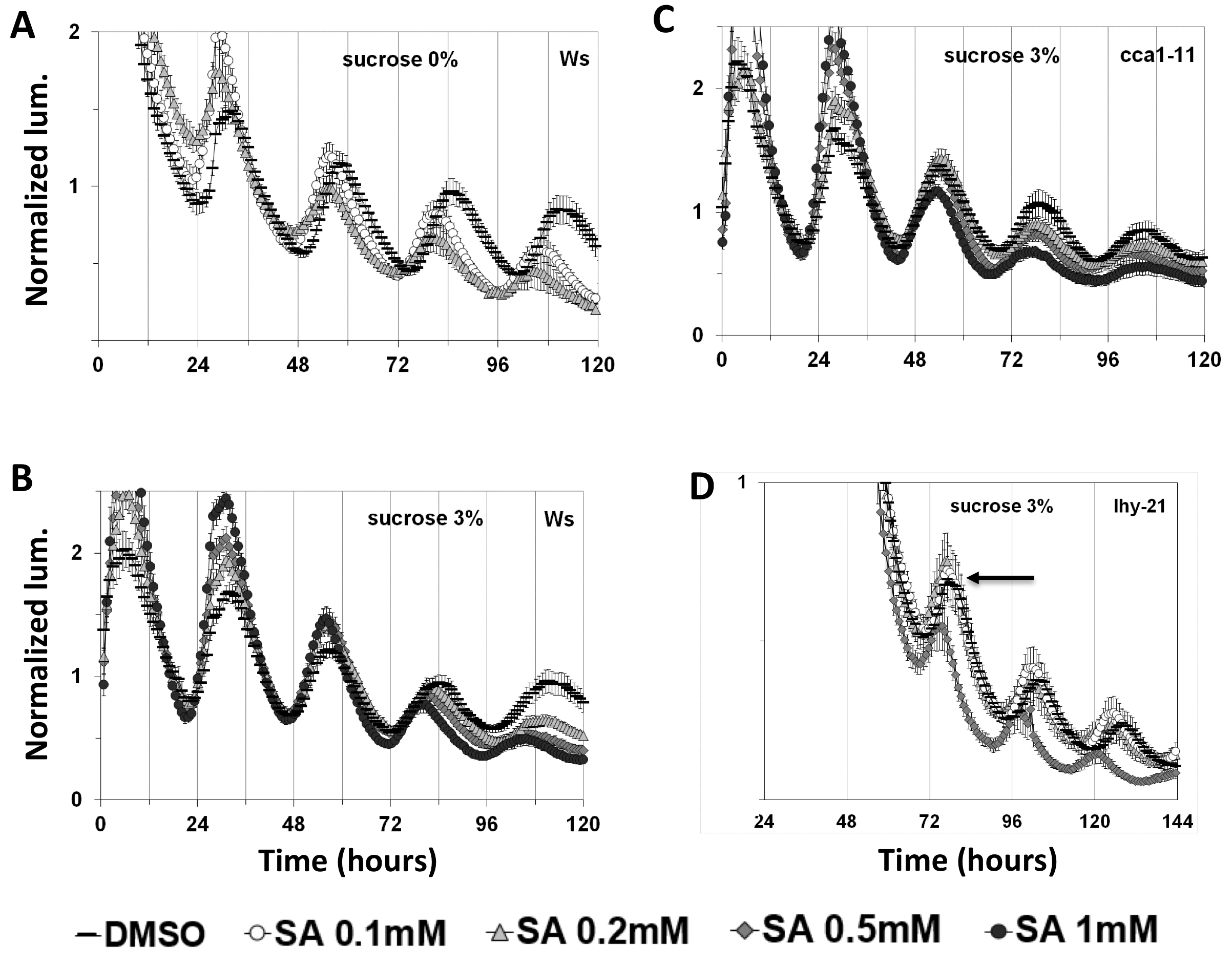

Supplement: FIGURE S4 — (A,B) Sucrose moderated the period-shortening effect of salicylic acid (SA) of the wild type harboring the CAB2:LUC transgene. (A,C) The cca1-11 mutant did not display any SA-related circadian phenotypes. (B,D) The lhy-21 mutant was slightly more sensitive than the wild type to SA-mediated peak advance [third peak shown with arrow in (D)]. Note that (D) does not display the first two peaks from zeitgeber time 0–48 h as in the other figures. Error bars represent standard error. [file Image_4.jpg]

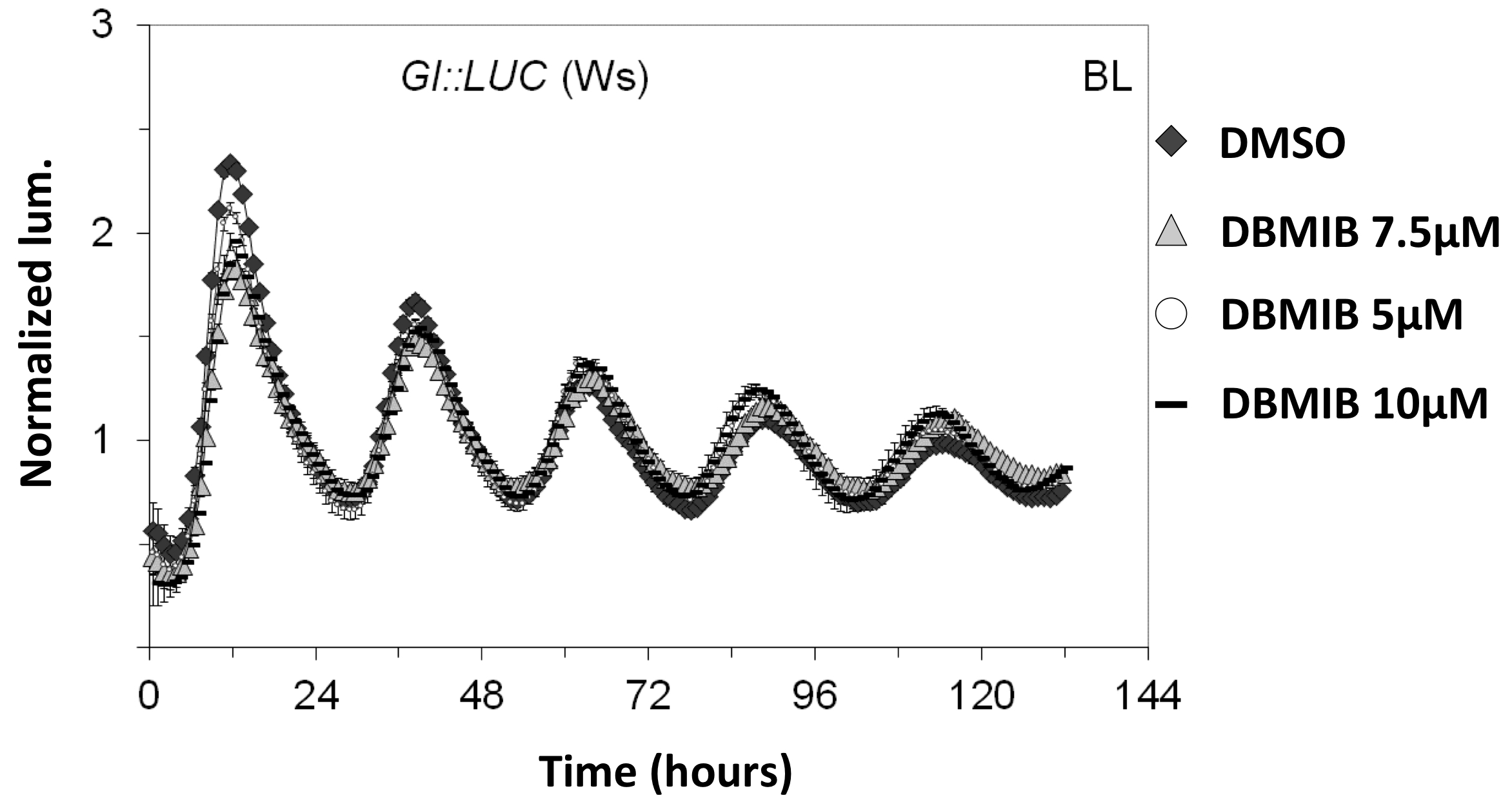

Supplement: FIGURE S5 — 2,5-Dibromo-3-methyl-6-isopropylbenzoquinone, unlike 3-(3,4-dichlorophenyl)-1,1-dimethylurea (Figure 1A), did not disturb the rhythmic expression of GI:LUC under blue light. The error bars are smaller than the symbols and represent standard error. [file Image_5.jpg]
