# Supplementary material for: Chemical Perturbation of Chloroplast-Related Processes Affects Circadian Rhythms of Gene Expression in Arabidopsis: Salicylic Acid Application Can Entrain the Clock
Source: Front Physiol. 2020 Jun 18;11:429. doi: 10.3389/fphys.2020.00429 (PMC7314985; doi:10.3389/fphys.2020.00429)
Supplement: Supplementary file 6 [file Data_Sheet_1.PDF]

Supplementary Table 1. Output from the ANOVA statistical analysis of **figure 1A.** Dose response of chemicals under on period constant darkness (left panel). Data was analyzed as indicated in material and methods. Briefly a one-way ANOVA with a post hoc Tukey test for multiple testing was used with SAS 9.0 package program. We used an ANOVA with two factors being these the chemicals (first factor) and their respective concentrations (second factor). Different letters indicate statistically significant different results.

**Treatment**	**# samples**	**Mean**	**Std Deviation**	**Tukey Group**
Rifampicin control	20	27.8895	1.23968789	B
Rifampicin 150 μg/mL	22	30.4777	1.41070712	A
SA control	17	27.4535	1.03448623	B
SA 1 mM	19	25.7963	0.67653702	C
Vit C control	24	28.1138	1.82503797	B
Vit C 2 mM	24	25.3095833	1.07917633	C
Vit C 3 mM	22	25.1091	1.10404843	C

Std - Standard deviation.

samples - indicate the number of individual seedlings tested.

Mean – Period mean value

Supplementary Table 2. Output from the ANOVA statistical analysis of **figure 1A.** Dose response of chemicals on period under red light (middle panel). Data was analyzed as indicated in material and methods and as in Supplementary Table 1. Different letters denote statistically significant different results.

**Treatment**	**# samples**	**Mean**	**Std Deviation**	**Tukey Group**
Vit C control	20	30.8874	2.07151271	A
Vit C 2 mM	22	27.6605	1.33112162	CD
DMCU control	17	27.5808	1.73865995	D
DMCU 5 μM	19	29.1604	2.05687073	BC
DMCU 10 μM	24	29.6800	1.63777288	AB

Std - Standard deviation

samples - indicate the number of individual seedlings tested.

Mean - Period mean value

Supplementary Table 3. Output from the ANOVA statistical analysis of **figure 1A.** Dose response of DMCU on period under blue light (right panel). Data was analyzed as indicated in material and methods and as in Supplementary Table 1 with the exception that a completely random test was applied instead of the two factors. Different letters indicate statistically significant different results.

**Treatment**	**# samples**	**Mean**	**Tukey Group**
DMCU control	24	26.5513	B
DMCU 5 μM	21	27.6629	AB
DMCU 7.5 μM	23	28.5852	A
DMCU 10 μM	24	28.4813	A

samples - indicate the number of individual seedlings tested.

Mean – Period mean value

Supplementary Table 4. Output from one-way ANOVA statistical analysis of **figure 1B** with *GI:LUC* as a gene reporter (left panel). Effect of paraquat (methylviologen) on circadian period. Data was analyzed as indicated in material and methods and as in Supplementary Table 1. The two factors considered were paraquat concentration and light quality. Different letters indicate statistically significant different results.

Treatment	# samples	Mean	Std Deviation	Tukey Group
Paraquat control Blue light	36	26.2078	1.27273184	C
Paraquat 7.5 μM control Blue light	30	27.5407	1.61755229	B
Paraquat 10 μM control Blue light	30	27.6750	1.83565783	B
Paraquat control Red light	23	29.2804	1.62989815	A
Paraquat 7.5 μM Red light	30	27.0400	1.72822013	BC
Paraquat 10 μM Red light	29	27.2510	2.28726653	BC

Std - Standard deviation

samples - indicate the number of individual seedlings tested.

Mean – Period mean value

Supplementary Table 5. Output from the ANOVA statistical analysis of **figure 1B** with *CCR2:LUC* as a gene reporter (right panel). Effect of paraquat on circadian period. Data was analyzed as indicated in material and methods and as in Supplementary Table 1. In this case the two factors also were paraquat concentration and light quality. Different letters indicate statistically significant different results.

Treatment	# samples	Mean	Std Deviation	Tukey Group
Paraquat control Blue light	41	26.0627	0.76879134	C
Paraquat 7.5 μM control Blue light	24	27.1717	1.19458076	B
Paraquat 10 μM control Blue light	17	28.1718	1.14738417	A
Paraquat control Red light	21	26.9290	1.01650826	BC
Paraquat 7.5 μM Red light	24	29.0221	1.63023866	A

Std - Standard deviation

samples - indicate the number of individual seedlings tested.

Mean – Period mean value

Supplementary Table 6A: The effect of SA on circadian period precision under RL+BL in the presence of supplementary sucrose 3%.

	DMSO			SA 0.5mM			SA 1mM		
	mean (h)	SD	SE	mean (h)	SD	SE	mean (h)	SD	SE
GI::LUC	26.90	1.42	0.17	25.66*	0.92	1.12	25.33*	0.72	0.09
CCA1::LUC	26.84	1.59	0.16	26.35	1.90	0.21	25.55*	0.70	0.08
TOC1::LUC	26.00	1.93	0.28	25.85	0.95	0.15	25.18	0.91	0.14
CCR2::LUC	26.92	1.11	0.10	26.22	0.85	0.08	26.46	0.63	0.06

Mean Period mean. SD Standard deviation. SE Standard Error. An asterisk denotes statistical significance ($p < 0.05$) for the SA-mediated period shortening (descriptive period - mean per). A simple pairwise comparison between the test population *versus* its control was performed for each comparison. The results show the combined data from several independent assays.

Supplementary Table 6B: The effect of SA on direct rhythmicity of rhythmic markers under RL+BL in the presence of supplementary sucrose 3%.

	DMSO		SA 0.5mM		SA 1mM	
	mean RAE	SE	mean RAE	SE	mean RAE	SE
GI::LUC	0.17	0.01	0.17	0.01	0.15*	0.01
CCA1::LUC	0.17	0.01	0.17	0.01	0.15*	0.01
TOC1::LUC	0.20	0.01	0.16*	0.01	0.11*	0.005
CCR2::LUC	0.22	0.01	0.19*	0.01	0.14*	0.004

Direct rhythmicity is inversely correlated to mean RAE. An asterisk indicates statistical significance ($p < 0.05$). A Student´s t-test was performed between the test population *versus* its control.

Supplementary Table 7. Output from the one-way ANOVA statistical analysis of **figure 2A** (*PHYB:LUC* under red light). Data was analyzed as indicated in material and methods and as in Supplementary Table 1. The factors in the analysis were sucrose and SA concentration respectively. Different letters denote statistically significant results.

Treatment	# samples	Mean	Std Deviation	Tukey Group
0 % sucrose / control SA	47	86.26	57.568284	C
0 % sucrose / SA 0.1 mM	48	128.79	86.487721	C
0 % sucrose / SA 0.5 mM	45	123.62	91.149300	C
3 % sucrose / control SA	46	185.80	154.031832	C
3 % sucrose / SA 0.1 mM	47	196.38	225.331211	C
3 % sucrose / SA 0.5 mM	47	468.62	493.117305	B
3 % sucrose / SA 1 mM	46	825.35	661.581916	A

Std - Standard deviation

samples - indicate the number of individual seedlings tested.

Mean – Luminescence mean value

Supplementary Table 8. Output from the ANOVA statistical analysis of **figure 2B** (*PHYB:LUC* under blue light). Data was analyzed as indicated in material and methods and as in Supplementary Table 1. The factors in the analysis were sucrose and SA concentration respectively. Different letters indicate statistically significant different results.

**Treatment**	**# samples**	**Mean**	**Std Deviation**	**Tukey Group**
0 % sucrose / control SA	41	150.9	57.568284	C
0 % sucrose / SA 0.1 mM	47	193.8	86.487721	C
0 % sucrose / SA 0.5 mM	44	234.2	91.149300	C
3 % sucrose / control SA	45	234.9	154.031832	C
3 % sucrose / SA 0.1 mM	47	380.8	225.331211	C
3 % sucrose / SA 0.5 mM	47	822.6	493.117305	B
3 % sucrose / SA 1 mM	47	1525.4	661.581916	A

Std - Standard deviation

samples - indicate the number of individual seedlings tested.

Mean – Luminescence mean value

Supplementary Table 9A: SA increased indirect rhythmicity of marker *PHYB::LUC* under RL and under BL.

light quality	SA concentration		
	0mM	SA 0.5Mm	SA 1mM
RL	a. 0.83±0.05	a. 0.54±0.06 ($p=5.24x10^{-4}$)	a. 0.26±0.02 ($p=7.88x10^{-12}$)
	b. 0.84±0.04	b. 0.63±0.06 ($p=4.75x10^{-3}$)	b. 0.42±0.06 ($p=7.54x10^{-7}$)
BL	c. 0.63±0.07	c. 0.42±0.07 (p=0.03)	c. 0.25±0.02 ($p=1.46x10^{-5}$)
	d. 0.67±0.06	d. 0.49±0.06 (p=0.04)	d. 0.45±0.06 (p=0.01)

Indirect rhythmicity is inversely correlated to mean RAE values shown. In parentheses p values represent the probability that mean RAE values are not affected by SA application. Results from two independent experiments for each light quality are shown.

Supplementary Table 9B: SA increased precision of marker *PHYB::LUC* under RL.

SA concentration	
0mM	SA 1mM
1.72 h<br>(1.89 h)	0.78 h<br>(1.13 h)
4.00<br>(4.14)	1.19 h<br>(1.28 h)

Precision is inversely correlated to SD of noPer and SD of period (not normalized, shown in parentheses). Results from two independent experiments are shown.

Supplementary Table 10. Output from the ANOVA statistical analysis of **figure 3A**. Effect of SA is gated and restricted to a specific time of the day. Data was analyzed as indicated in material and methods and as in Supplementary Table 1. A totally random experimental design was applied for the ANOVA analysis. Different letters indicate statistically significant different results.

**Treatment**	**# samples**	**Mean**	**Tukey Group**
Darkness	93	27.6244	A
0-3 ctrl	74	26.6027	ABCD
0-3 SA 1 mM	69	25.6071	D
3-6 ctrl	70	26.2351	BCD
3-6 SA 1 mM	70	25.7596	CD
6-9 ctrl	78	26.9536	AB
6-9 SA 1 mM	76	26.7901	ABC
9-12 ctrl	71	26.3231	BCD
9-12 SA 1 mM	47	26.3381	BCD

samples - indicate the number of individual seedlings tested.

Mean – Period mean value

Supplementary Table 11: The effect of SA on precision of *CCR2::LUC* is correlated to parametric entrainment.

days of entrainment events	SD of noPer (h)		
	DMSO	SA 0.5mM	SA 1mM
0	1.36	0.72	0.75
1	1.16	0.84	0.48
2	2.10	1.13	0.78
3	2.20	1.36	0.67

SD of noPer (inversely correlated to precision) is increased by entrainment in 96-well microtiter plates. This effect is attenuated by SA application. Oscillations are shown in in Supplementary Figure 2A.

Supplementary Table 12: Entrainment of seedlings in 96-well microtiter plates decreases indirect rhythmicity of *CCR2::LUC* oscillations. This effect is attenuated by SA application.

SA (mM)	days of entrainment events				Z (p value)
	0 days	1 day	2 days	3 days	
0 mM	a. 0.26±0.02	a. 0.46±0.06 (0.01)	a. 0.62±0.07 ($2.18x10^{-5}$)	a. 0.68±0.07 ($1.10x10^{-5}$)	
	b. 0.19±0.01	b. 0.22±0.01 (0.01)	b. 0.51±0.03 ($3.86x10^{-16}$)	b. 0.52±0.03 ($2.63x10^{-16}$)	
	c. 0.20±0.02	c. np	c. np	c. 0.34±0.04 ($7.78x10^{-4}$)	
0.2 mM	a. 0.21±0.01	a. 0.40±0.05 ($1.03x10^{-3}$)	a. 0.35±0.01 ($6.81x10^{-9}$)	a. 0.44±0.05 ($3.21x10^{-4}$)	a. $6.81x10^{-19}$
0.5 mM	a. 0.22±0.02	a. 0.37±0.05 ($3.29x10^{-3}$)	a. 0.37±0.05 ($6.75x10^{-3}$)	a. 0.35±0.05 (0.01)	a. $5.09x10^{-41}$
	b. 0.20±0.01	b. 0.23±0.01 (0.08)	b. 0.28±0.01 ($1.73x10^{-6}$)	b. 0.27±0.01 ($1.42x10^{-6}$)	b. ~0
	c. 0.15±0.01	c. np	c. np	c. 0.39±0.06 ($2.67x10^{-4}$)	c. nr
1 mM	a. 0.21±0.02	a. 0.27±0.01 (0.01)	a. 0.26±0.01 (0.01)	a. 0.26±0.01 (0.04)	a. $7.37x10^{-77}$
	b. 0.15±0.01	b. 0.17±0.01 (0.04)	b. 0.22±0.01 ($2.09x10^{-9}$)	b. 0.25±0.01 ($6.79x10^{-17}$)	b. ~0
	c. 0.14±0.01	c. np	c. np	c. 0.20±0.01 ($3.23x10^{-6}$)	c. $2.84x10^{-32}$

Entrainment of seedlings in 96-well microtiter plates decreases indirect rhythmicity (inversely correlated to mean RAE shown here). p values in parentheses represent the probability that mean RAE is not increased by entrainment. The effect of entrainment on indirect rhythmicity is attenuated by SA application. Z represents the probability (p value) that SA application does not attenuate this effect after 3 days of entrainment. Results from three independent experiments (a, b and c) are shown. np is for not performed experiment. nr is for negative results.

Supplementary Table 13: The effect of SA on rhythmicity of *CCR2::LUC* expression is enhanced by parametric entrainment.

SA concentration	MeanΔRAE(0) for 0 additional entrainment events	MeanΔRAE(3) for 3 additional entrainment events	Z (p value)
0.2mM	a. 0.05±0.01	a. 0.23±0.02	a. $1.51x10^{-20}$
0.5mM	a. 0.04±0.01	a. 0.32±0.02	a. $7.05x10^{-43}$
	b. -0.01±0.001	b. 0.25±0.01	b. ~0
	c. 0.05±0.004	c. -0.05±0.01	c. nr
1mM	a. 0.05±0.01	a. 0.42±0.02	a. $1.99x10^{-88}$
	b. 0.04±0.001	b. 0.27±0.005	b. ~0
	c. 0.06±0.004	c. 0.15±0.01	c. $2.93x10^{-28}$

The effect of SA on indirect rhythmicity MeanΔRAE after 0 and 3 days of entrainment is shown. It was calculated as the mean of the differences (in RAE values) produced when individual plants that received 0 mM (DMSO solvent) and one of the indicated SA concentrations were paired. Z is the probability (p value) that meanΔRAE(0) is equal to meanΔRAE(3). Pairwise comparisons were done with a Student´s t –test. Results from three independent experiments (a, b and c) are shown. SA caused a substantial decrease in meanΔRAE (increase of indirect rhythmicity) only when additional entrainment events were applied. nr is for negative results.

Supplementary Table 14: The effect of SA on phase of *CCR2::LUC* expression is enhanced by parametric entrainment.

SA concentration	meanΔphase(0) for 0 additional entrainment events (h)	meanΔphase(3) for 3 additional entrainment events (h)	Z (p value)
0.2mM	a. 2.03±0.17	a. 2.91±0.13	a. 6.62×10^{-05}
0.5mM	a. 2.10±0.16	a. 3.19±0.14	a. 1.97×10^{-07}
	b. 2.44±0.04	b. 3.77±0.06	b. 2.61×10^{-72}
	c. 0.35±0.06	c. 3.83±0.14	c. 9.70×10^{-101}
1mM	a. 1.40±0.16	a. 4.91±0.11	a. 6.12×10^{-61}
	b. 1.84±0.04	b. 5.06±0.06	b. ~0
	c. -1.33±0.07	c. 4.56±0.12	c. 2.30×10^{-244}

The effect of SA (meanΔphase) on phase (timing of the second circadian peak) of *CCR2::LUC* is enhanced by parametric entrainment. Results from three independent experiments (a, b and c) are shown. P value obtained after a Student´s t-test for each pair of populations tested.

Supplementary Table 15: The effect of SA on phase of *GI::LUC* expression is enhanced by parametric entrainment.

SA concentration	meanΔphase for 0 additional entrainment events (h)	meanΔphase for 3 additional entrainment events (h)	meanΔphase for 7 additional entrainment events (h)	p value
0.1mM	b. 1.43±0.20	b. np	b. 3.96±0.18	b. $2.85x10^{-20}$
0.2mM	a. 1.16±0.20	a. 4.86±0.13	np	a. $8.80x10^{-49}$
	b. 1.70±0.22	np	b. 4.01±0.19	b. $5.06x10^{-15}$
0.5mM	a. 0.52±0.0.19	a. 6.13±0.14	np	a. $4.63x10^{-95}$
	b. 1.22±0.20	np	b. 6.12±0.17	b. $6.02x10^{-68}$
	c. 1.17±0.10	c. 3.71±0.16	np	c. $7.37x10^{-38}$
1mM	a. 2.36±0.0.17	a. 8.46±0.12	np	a. $9.60x10^{-126}$
	c. 1.50±0.09	c. 5.49±0.13	np	c. $1.10x10^{-116}$

The effect of SA (meanΔphase) on phase (timing of the second circadian peak) of *GI::LUC* is enhanced by parametric entrainment. meanΔphase is calculated as described for meanΔRAE in Supplementary Table 3d. p values represent the probability that meanΔphase(0) is equal to meanΔphase(3). Results from three independent experiments (a, b and c) are shown. P value obtained after a Student´s t-test for each pair of populations tested. np is for not performed experiment.

Supplementary Table 16. Output from the one-way ANOVA statistical analysis of **figure 4A** (*GI:LUC* in Ws background under blue light). Data was analyzed as indicated in material and methods and as in Supplementary Table 1. The two factors considered in the experimental design were sucrose concentration and SA concentration. Different letters indicate statistically significant different results.

Treatment	# samples	Mean	Std Deviation	Tukey Group
0 % sucrose / control	94	27.0062	1.90791453	A
0 % sucrose / SA 0.1 mM	67	25.5679	1.82261928	BC
0 % sucrose / SA 0.2 mM	46	24.2287	1.35162061	DE
0 % sucrose / SA 0.5 mM	68	23.9237	1.75250942	E
3 % sucrose / control	71	26.3128	1.30579607	AB
3 % sucrose / SA 0.1 mM	24	26.3588	0.92294572	AB
3 % sucrose / SA 0.5 mM	48	25.5965	0.82128464	BC
3 % sucrose / SA 0.75 mM	24	24.9471	1.10421122	CD
3 % sucrose / SA 1 mM	71	25.4587	1.16968974	BC

Std - Standard deviation

samples - indicate the number of individual seedlings tested.

Mean - Period mean value

Supplementary Table 17. Output from the ANOVA statistical analysis of **figure 4B** (*GI:LUC* in Ws background under red light). Data was analyzed as indicated in material and methods and as in Supplementary Table 1. SA and sucrose concentration were the two factors considered in the test. Different letters indicate statistically significant different results.

Treatment	# samples	Mean	Std Deviation	Tukey Group
0 % sucrose / control	102	31.1746	1.88585704	A
0 % sucrose / SA 0.1 mM	93	28.6311	1.95484430	BCD
0 % sucrose / SA 0.2 mM	49	27.6678	2.52950939	D
0 % sucrose / SA 0.5 mM	45	25.5887	1.27134610	E
3 % sucrose / control	48	29.8202	2.13459996	B
3 % sucrose / SA 0.1 mM	21	29.5857	2.04194655	BC
3 % sucrose / SA 0.5 mM	23	28.3361	1.59575214	CD
3 % sucrose / SA 1 mM	47	26.3391	0.74246894	E

Std - Standard deviation

samples - indicate the number of individual seedlings tested.

Mean - Period mean value

Supplementary Table 18. Output from the ANOVA statistical analysis of **figure 4C** (*GI:LUC* in Col-0 background under blue light). Data was analyzed as indicated in material and methods and as in Supplementary Table 1. The two factors considered in the experimental design were sucrose concentration and SA concentration. Different letters indicate statistically significant different results.

Treatment	# samples	Mean	Std Deviation	Tukey Group
0 % sucrose / control	208	27.5928	2.03380611	A
0 % sucrose / SA 0.1 mM	185	26.0459	1.37712263	B
0 % sucrose / SA 0.2 mM	186	25.5969	1.47394303	BC
0 % sucrose / SA 0.5 mM	70	24.6477	1.66042178	D
3 % sucrose / control	71	25.3041	0.92035176	CD
3 % sucrose / SA 0.2 mM	48	25.3700	0.79116397	BC
3 % sucrose / SA 0.5 mM	72	25.3156	0.61335579	CD
3 % sucrose / SA 1 mM	70	25.2733	0.79880839	CD

Std - Standard deviation

samples - indicate the number of individual seedlings tested.

Mean - Period mean value

Supplementary Table 19. Output from the statistical analysis of **figure 2E** (red light). Effect of SA on phase advance. Data was analyzed as indicated in material and methods and as in Supplementary Table 1. The two factors for the analysis were SA concentration and genotype. Different letters indicate statistically significant different results.

**Treatment**	**# samples**	**Mean**	**Std Deviation**	**Tukey Group**
wt control	95	105.8248	4.45345954	A
wt / SA 0.1 mM	83	102.3166	4.29228130	B
wt / SA 0.5 mM	86	101.3781	3.31459323	BC
phyB-9 control	73	102.0214	3.12672633	B
phyB-9 / SA 0.1 mM	72	100.9233	3.06284964	BC
phyB-9 / SA 0.5 mM	61	100.1967	3.67167842	C

Std - Standard deviation

samples - indicate the number of individual seedlings tested.

Mean - Phase mean value (third peak)

Supplementary Table 20. Output from the statistical analysis of **figure 2F** (blue light). Effect of SA on clock period in the mutant *phyB-9*. Data was analyzed as indicated in material and methods and as in Supplementary Table 1. The two factors for the analysis were SA concentration and genotype. Different letters indicate statistically significant results.

Treatment	# samples	Mean	Std Deviation	Tukey Group
wt control	130	27.3935	1.90148733	A
wt / SA 0.1 mM	111	25.9385	1.17407464	B
wt / SA 0.2 mM	110	25.5526	1.55641005	B
wt / SA 0.5 mM	23	23.4261	1.43021530	C
phyB-9 control	101	26.4587	1.53082831	AB
phyB-9 / SA 0.1 mM	81	26.1026	1.48439784	B
phyB-9 / SA 0.2 mM	83	25.7466	1.96539407	B
phyB-9 / SA 0.5 mM	18	23.1661	1.82311027	C

Std - Standard deviation

samples - indicate the number of individual seedlings tested.

Mean - Period mean value

Supplementary Table 21. Output from the statistical analysis of **figure 5B**. Effect of SA circadian clock periodicity of *toc1-21* is dependent on sucrose supplementation. Data was analyzed as indicated in material and methods and as in Supplementary Table 1. The two factors for the ANOVA analysis were sucrose concentration and genotype. Different letters indicate statistically significant results.

Treatment	# samples	Mean	Std Deviation	Tukey Group
wt / 0 % sucrose	35	32.9291	3.70430463	A
toc1-21 / 0% sucrose	47	32.2753	2.66753334	A
wt / 3 % sucrose	48	27.6652	1.40462305	B
toc1-21 / 0% sucrose	43	24.2477	1.65632387	C

Std - Standard deviation

samples - indicate the number of individual seedlings tested.

Mean - Period mean value

Supplementary Table 22. Output from the one-way ANOVA statistical analysis of **figure 5C.** Effect of SA application on circadian clock periodicity of *toc1-21* under red light in media without sucrose. Data was analyzed as indicated in material and methods and as in Supplementary Table 1. The two factors for the ANOVA analysis were SA concentration and genotype. Different letters indicate statistically significant results.

**Treatment**	**# samples**	**Mean**	**Std Deviation**	**Tukey Group**
wt control	67	29.6682	1.40871844	B
toc1-21 control	57	33.0946	4.93967866	A
wt SA 0.1 mM	55	27.1785	1.15583290	C
toc1-21 0.1 mM	52	27.8656	1.97697175	C

Std - Standard deviation

samples - indicate the number of individual seedlings tested.

Mean - Period mean value

Supplementary Table 23. Output from the statistical analysis of **figure 5D.** Effect of SA application on circadian clock periodicity of *toc1-21* under blue light without sucrose. Data was analyzed as indicated in material and methods. Genotype and SA concentration were the two factors of the analysis. Different letters indicate statistically significant results.

Treatment	# samples	Mean	Std Deviation	Tukey Group
wt control	24	25.9358	1.49814644	A
toc1-21 control	22	21.3300	1.89857465	C
wt SA 0.1 mM	15	24.0755	1.19709461	B
toc1-21 0.1 mM	21	21.2943	1.53188306	C

Std - Standard deviation

samples - indicate the number of individual seedlings tested.

Mean - Period mean value

Supplementary Table 24. Output from the one-way ANOVA statistical analysis with an experimental design of two factors of **figure 6A.** *gi-11* mutant *PHYB:LUC* luminescence under red light on media without sucrose. Data was analyzed as indicated in material and methods and as in Supplementary Table 1. The two factors considered for the ANOVA analysis were SA concentration and genotype. Different letters indicate statistically significant results.

Treatment	# samples	Mean	Std Deviation	Tukey Group
wt / SA control	93	55.369	32.406050	D
wt / SA 0.1 mM	93	73.172	28.514355	CD
wt / SA 0.2 mM	93	85.620	29.849540	C
wt / SA 0.5 mM	93	95.156	28.746900	C
gi-11 / SA control	93	98.701	46.067143	C
gi-11 / SA 0.1 mM	93	201.836	86.504971	B
gi-11 / SA 0.2 mM	93	210.273	84.601307	B
gi-11 / SA 0.5 mM	93	239.893	103.953687	A

Std - Standard deviation

samples - indicate the number of individual seedlings tested.

Mean - Luminescence mean value

Supplementary Table 25. Output from the ANOVA statistical analysis of **figure 6B**. *gi-11* mutant *PHYB:LUC* luminescence under blue light on media without sucrose. Data was analyzed as indicated in material and methods and as in Supplementary Table 1. The two factors for the ANOVA analysis were SA concentration and genotype. Different letters indicate statistically significant results.

Treatment	# samples	Mean	Std Deviation	Tukey Group
wt / SA control	93	88.20	19.791594	F
wt / SA 0.1 mM	93	100.55	12.850294	F
wt / SA 0.2 mM	93	152.43	14.814347	E
wt / SA 0.5 mM	93	193.24	25.126210	D
gi-11 / SA control	93	133.95	36.495876	E
gi-11 / SA 0.1 mM	93	263.22	40.211671	C
gi-11 / SA 0.2 mM	93	403.91	68.230033	B
gi-11 / SA 0.5 mM	93	933.40	180.721712	A

Std - Standard deviation

samples - indicate the number of individual seedlings tested.

Mean - Luminescence mean value

Supplementary Table 26. Output from the ANOVA statistical analysis of **figure 6C.** *lhy-21 CAB2:LUC* luminescence under blue light. Data was analyzed as indicated in material and methods and as in Supplementary Table 1. The two factors for the ANOVA analysis were SA concentration and genotype. Different letters indicate statistically significant results.

Treatment	# samples	Mean	Std Deviation	Tukey Group
wt / SA control	93	82.1643	2.10658052	A
wt / SA 0.1 mM	47	81.5178	1.81984164	A
wt / SA 0.2 mM	94	81.4445	1.84206325	A
wt / SA 0.5 mM	96	81.0117	2.08153216	AB
wt / SA 1.0 mM	24	80.0767	1.70439500	B
lhy-21 / SA control	92	78.6153	2.18011818	C
lhy-21 / SA 0.1 mM	46	76.2846	2.07500437	D
lhy-21 / SA 0.2 mM	83	76.2545	2.67206701	D
lhy-21 / SA 0.5 mM	87	73.9378	2.18190200	E
lhy-21 / SA 1.0 mM	40	72.5153	1.99730329	F

Std - Standard deviation

samples - indicate the number of individual seedlings tested.

Mean - Luminescence mean value

Supplementary Table 27: The *lhy-21* mutant is more sensitive than wild type to SA-mediated peak advance of rhythmic marker *CAB2::LUC*.

	SA concentration			
	0.1mM	0.2mM	0.5m	1mM
X = p value for *Ws*	0.08	0.78	7.65×10^{-2}	8.11×10^{-4}
Y = p value for *lhy-21*	2.18×10^{-8}	2.03×10^{-9}	2.02×10^{-31}	3.38×10^{-26}
Z	~0	~0	~0	~0

X, Y represent the probability (p value) that SA does not advance the timing of the 3rd circadian peak in the wild type and in the *lhy-21* mutant respectively. Z is the probability that the effect of SA on the timing of the third circadian peak is equal in the mutant and in the wild type. X, Y and Z refer to descriptive data (not RAE-normalized data). P-values obtained from a pairwise comparison between the indicated treatment *versus* the control. Four independent experiments produced similar results. Combined data from these experiments are shown.
